# Synthesis, Docking, 3-D-Qsar, and Biological Assays of Novel Indole Derivatives Targeting Serotonin Transporter, Dopamine D2 Receptor, and Mao-A Enzyme: In the Pursuit for Potential Multitarget Directed Ligands

**DOI:** 10.3390/molecules25204614

**Published:** 2020-10-10

**Authors:** Christopher Cerda-Cavieres, Gabriel Quiroz, Patricio Iturriaga-Vásquez, Julio Rodríguez-Lavado, Jazmín Alarcón-Espósito, Claudio Saitz, Carlos D. Pessoa-Mahana, Hery Chung, Ramiro Araya-Maturana, Jaime Mella-Raipán, David Cabezas, Claudia Ojeda-Gómez, Miguel Reyes-Parada, Hernán Pessoa-Mahana

**Affiliations:** 1 Departamento de Química Orgánica y Fisicoquímica, Facultad de Ciencias Químicas y Farmacéuticas, Universidad de Chile, Casilla 233, Santiago 8380494, Chile; cdc.cavieres@gmail.com (C.C.-C.); julio.rodriguez@ciq.uchile.cl (J.R.-L.); jazmin.alarc@gmail.com (J.A.-E.); clsaitz@ciq.uchile.cl (C.S.); 2 Programa de Doctorado en Farmacología, Universidad de Chile, Santiago 8380453, Chile; gquirozn@ciq.uchile.cl; 3 Departamento de Ciencias Químicas y Recursos Naturales, Facultad de Ingeniería y Ciencias, Universidad de la Frontera, Temuco 4811230, Chile; patricio.iturriaga@ufrontera.cl; 4 Departamento de Farmacia, Facultad de Química, Pontificia Universidad Católica de Chile, Santiago 7820244, Chile; cpessoa@puc.cl (C.D.P.-M.); chung.hery@gmail.com (H.C.); 5 Instituto de Química y Recursos Naturales, Universidad de Talca, Talca 3460000, Chile; raraya@utalca.cl; 6 Instituto de Química y Bioquímica, Facultad de Ciencias, Universidad de Valparaíso, Av. Gran Bretaña 1111, Valparaíso 2360102, Chile; jaime.mella@uv.cl (J.M.-R.); jaime.mella@uc.cl (D.C.); 7 Centro de Investigación Farmacopea Chilena (CIFAR), Universidad de Valparaíso, Av. Gran Bretaña 1111, Valparaíso 2360102, Chile; 8 Colegio Instituto San Martín, Hermanos Maristas, Curicó 3341965, Chile; claudia-ojeda@gmail.com; 9 Centro de Investigación Biomédica y Aplicada (CIBAP), Escuela de Medicina, Facultad de Ciencias Médicas, Universidad de Santiago de Chile, Santiago 9170002, Chile; 10 Facultad de Ciencias de la Salud, Universidad Autónoma de Chile, Talca 3467987, Chile

**Keywords:** polypharmacology, SERT, dopamine D_2_ receptor, 3-indolylpropylpiperazines, multitarget, docking, QSAR

## Abstract

A series of 27 compounds of general structure 2,3-dihydro-benzo[1,4]oxazin-4-yl)-2-{4-[3-(1*H*-3indolyl)-propyl]-1-piperazinyl}-ethanamides, Series I: **7**(**a**–**o**) and (2-{4-[3-(1*H*-3-indolyl)-propyl]-1-piperazinyl}-acetylamine)-*N*-(2-morfolin-4-yl-ethyl)-fluorinated benzamides Series II: **13**(**a**–**l**) were synthesized and evaluated as novel multitarget ligands towards dopamine D_2_ receptor, serotonin transporter (SERT), and monoamine oxidase-A (MAO-A) directed to the management of major depressive disorder (MDD). All the assayed compounds showed affinity for SERT in the nanomolar range, with five of them displaying *K*i values from 5 to 10 nM. Compounds **7k**, *K*i = 5.63 ± 0.82 nM, and **13c**, *K*i = 6.85 ± 0.19 nM, showed the highest potencies. The affinities for D_2_ ranged from micro to nanomolar, while MAO-A inhibition was more discrete. Nevertheless, compounds **7m** and **7n** showed affinities for the D_2_ receptor in the nanomolar range (**7n**: *K*i = 307 ± 6 nM and **7m**: *K*i = 593 ± 62 nM). Compound **7n** was the only derivative displaying comparable affinities for SERT and D_2_ receptor (D_2_/SERT ratio = 3.6) and could be considered as a multitarget lead for further optimization. In addition, docking studies aimed to rationalize the molecular interactions and binding modes of the designed compounds in the most relevant protein targets were carried out. Furthermore, in order to obtain information on the structure–activity relationship of the synthesized series, a 3-D-QSAR CoMFA and CoMSIA study was conducted and validated internally and externally (q^2^ = 0.625, 0.523 for CoMFA and CoMSIA and r^2^_ncv_ = 0.967, 0.959 for CoMFA and CoMSIA, respectively).

## 1. Introduction

Major depressive disorder (MDD) is a common, chronic, recurring, heterogeneous, and potentially life-threatening disease affecting up to 20% of the world population, according to World Health Organization [1]. MDD is triggered by a complex pattern of genetic, epigenetic, and environmental factors and, despite its prevalence, it is not being properly treated [2,3,4]. As a result, such a devastating disorder is enormously costing, economically, socially, and individually [5,6].

Since the monoamine hypothesis of depression was developed in the 1950s [7], antidepressant drug discovery has been a very active research field [8], although MDD’s underlying mechanism and neurological basis are not yet fully understood [9,10,11]. The global antidepressants market is expected to grow from $14.3 billion in 2019 to about $28.6 billion in 2020 as mental health issues are expected to surge because of the Covid-19 pandemic making an impact on the global economy.

Thus far, the vast majority of marketed antidepressants act through the serotoninergic and norepinephrinergic systems, elevating synaptic levels of the corresponding neurotransmitters by blocking the monoamine transporters [12,13,14,15]. Many adverse effects associated with first-generation antidepressants (mainly tricyclic and monoamine oxidase inhibitors, –MAOi–) were partially overcome by the arrival of second-generation antidepressants, including selective serotonin reuptake inhibitors (SSRIs), norepinephrine reuptake inhibitors (NRIs), and dual antidepressants (SNRIs), with the latter being advantageous as they can treat a wide range of symptoms [16,17,18,19]. However, much less approved antidepressants do target the dopamine system, notwithstanding dopamine has also been implicated in the pathophysiology of depression [20].

Inside the central nervous system (CNS), dopamine plays pivotal roles in executive functions, motor control, emotion, cognition, reinforcement, and reward, among others [21,22,23]. Its dysfunction is related to the pathophysiology of several CNS disorders, such as Parkinson’s disease, multiple sclerosis, schizophrenia, drug addiction, and MDD [24,25,26]. Even though depression has been historically associated with misregulation within the serotonin/norepinephrine system, in the last years, an increasing number of voices have been raised supporting that dopamine system dysfunction strings along with it [24,27,28].

Due to the aforementioned causes, MDD is nowadays recognized as a complex dynamic system [29] endowed with a complex pathophysiology. For this reason, depression symptoms and its underlying causes are more likely to be treated by a multitarget approach, by using promiscuous drugs also defined as multi-target directed ligands (MTDLs): A single drug molecule that selectively targets multiple receptors, so that different pathways conducting to the disease can be modified by using a single molecule [20,30,31,32].

Indole derivatives have always been recognized as serotoninergic modulators, given their related structural connection. Furthermore, in our research group, we have extensive experience in the synthesis of indolylpropyl-piperazine derivatives, which have been successfully employed by us [33,34,35,36,37] and other groups [38,39] as very potent serotonin transporter (SERT) ligands. On the other hand, molecular structures containing morpholine [40] or benzoxazinone [41,42,43,44,45] cores have been reported as MAOi or dopamine D_2_ receptor modulators, respectively. In the light of such a background, we decided to conduct an exploratory study by merging the aforementioned indolylpropyl-piperazines and morpholine/benzoxazinone units. Herein, we describe the synthesis, docking, QSAR, and biological evaluation of two series of molecular hybrids targeting SERT, MAO-A, and D_2_ receptor in the pursuit of promising multi-target leads for potential MDD treatment.

## 2. Results and Discussion

### 2.1. Chemistry Series I

#### 2,3-Dihydro-benzo[*b*][1,4] oxazin-4-yl)-2-{4-[3-(1*H*-3-indolyl)-propyl]-1-piperazinyl} ethanamide derivatives **7a**–**o**

**Scheme 1:**
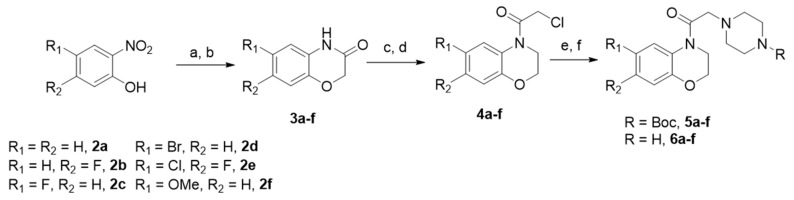
Synthesis of piperazinylbenzoxazine derivatives **6** (**a**–**c**). *Reagents and experimental conditions*. (a) Fe°/(H_2_O/EtOH/CH_3_COOH) 1:1:1/60 °C; (b) 2-chloroacetyl chloride, CH_3_CN, K_2_CO_3_, 80 °C; (c) THF/LiAlH_4_/N_2_/0 °C; (d) 2-chloroacetyl chloride-THF, 0 °C; (e) *N*-Boc-piperazine, CH_3_CN, K_2_CO_3_, 80 °C; (f) TFA- CH_2_Cl_2_, 0 °C.

**Scheme 2:**
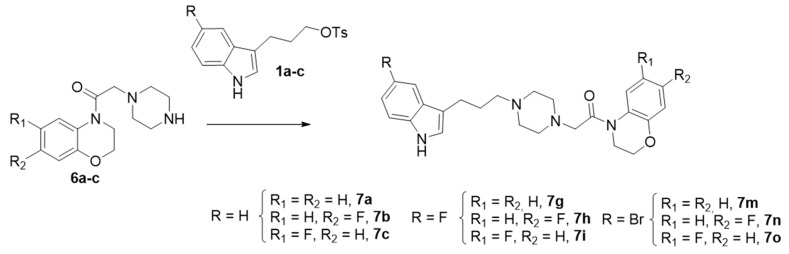
Synthesis of Series I. *Reagents and conditions*: K_2_CO_3_, CH_3_CN, 80 °C, Yield (42–89%).

The synthetic approach for the preparation of the target compounds **7a**–**o** is outlined below. The reaction took place between piperazine benzoxazine derivatives **6**(**a**–**c**) obtained in a six-step sequence and 82–95% yields (Scheme 1), with 3-indolyl tosylates **1a**–**c** (R = H, F, Br) obtained by reported literature procedures [37,46] to give nine final compounds **7**(**a**, **b**, **c**, **g**, **h**, **i**, **m**, **n**, **o**) in a 42–89% yield (Scheme 2).

**Scheme 3:**
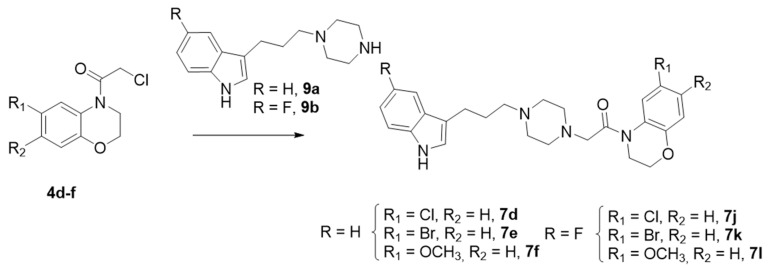
Synthesis of Series I. Route 2. *Reagents and conditions*: K_2_CO_3_, CH_3_CN, 80 °C, Yield (63–91%).

The synthesis of the derivatives **7d**–**f** and **7j**–**l** was accomplished by a different synthetic strategy, involving indolylpiperazines **9a**–**b** [35], which were reacted with *N*-chloroacetyl benzoxazines **4d**–**f** and **4j**–**l** to give the aforementioned six final compounds (**7d**–**f** and **7j**–**l**) with good to excellent yields (Scheme 3).

In summary, 15 compounds were synthesized for Series I in yields ranging from 42% to 91%.

### 2.2. Chemistry Series II

#### (2-{4-[3-(1*H*-Indol-3-yl)-propyl]-1-piperazinyl}-acetylamine)-*N*-(2-morpholin-4-yl-ethyl)-fluorinated benzamides

**Scheme 4:**
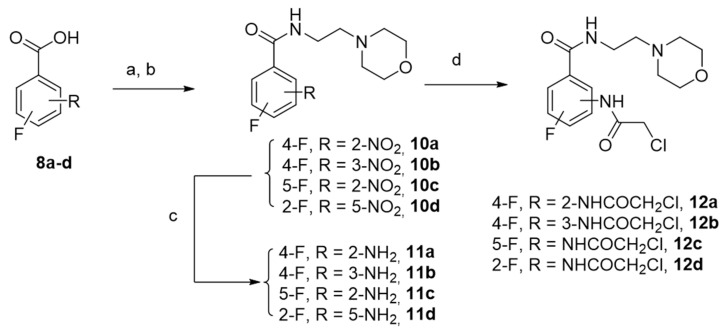
Synthesis of derivatives **12a**–**d**. *Reagents and experimental conditions*: (a) SOCl_2_, 60 °C; (b) 2-aminoethyl morpholine, anhydrous THF; (c) Fe°/(H_2_O/EtOH/CH_3_COOH)/60°C; (81–98%) (d) 2-chloroacetylchloride, anhydrous THF, 0 °C, Yield (93–97%).

The synthetic pathway of this series involved the fluorinated benzamide derivatives **12a**–**d**, which were obtained from commercially available isomeric fluoro nitrobenzoic acids in a three-step sequence with good to excellent yields as shown in Scheme 4.

**Scheme 5:**
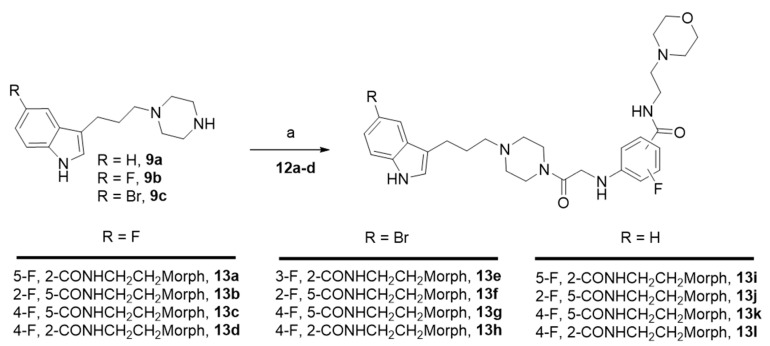
Synthesis of Series II derivatives **13a**–**l**. *Reagents and conditions*: K_2_CO_3_, CH_3_CN, 80 °C, Yield (47–85%).

The fluorinated benzamides derivatives **12a**–**d** were finally connected to indolylpropylpiperazines **9a**–**c** to achieve the expected compounds **13a**–**l**, with yields ranging from 47% to 85% (Scheme 5).

### 2.3. Pharmacology Series I

Table 1 summarizes the affinity of compounds **7a**–**o** for SERT, D_2_ receptor, and MAO-A. Most compounds were potent and clearly selective as SERT ligands, showing in all cases affinities in the nanomolar range, whereas the affinities for D_2_ and MAO-A ranged from micromolar to much higher values, respectively. A detailed analysis of SERT activities indicates that a C-5 substitution of the indole ring with halogens (fluorine or bromine; compounds **7g** or **7m**) leads to more potent compounds than the unsubstituted derivative (**7a**). On the other hand, the presence of a halogen atom at C-6 of the benzoxazine ring increased the affinity (e.g., **7c**, **7d**, and **7e** vs. **7a**). Accordingly, the most potent compounds were those exhibiting a dual halogen substitution pattern (**7i**, **7j**, and **7k**), with Ki values below 10 nM. The C-7 halogen substitution on the benzoxazine ring gave no consistent effects, slight increases (**7b** and **7h**) or decreases (**7n**) of affinity were observed, as compared with the corresponding C-7 unsubstituted compounds (**7a**, **7g**, and **7m**, respectively). The D_2_ receptor affinity for this series indicates that no conclusive structure–activity relationships can be extracted for these compounds. Nevertheless, it is apparent that dihalogenated derivatives, bearing one halogen atom at the C-5 of the indole ring and the other at either the C-6 or C-7 of the benzoxazine moiety (**7h**–**7k**, **7m**–**7o**), resulted in more potent compounds than their corresponding monohalogenated or unsubstituted counterparts (**7a**–**7e**, **7g**). Moreover, the presence of a methoxyl group at the C-6 of the benzoxazine ring has almost no effect on the affinity of the compounds for D_2_ receptor. It is worth mentioning that the dihalogenated compound **7n** was the only derivative displaying comparable affinities for SERT and D_2_ receptor (D_2_/SERT ratio = 3.6) and could be considered as a potential leader in the search of more potent multitarget compounds.

### 2.4. Docking Simulation Series I

Considering the pharmacological results, docking studies aimed to rationalize the molecular interactions and binding modes of the designed compounds were carried out only in the human SERT (hSERT) and in selected cases at the D_2_ receptor.

#### 2.4.1. hSERT

**Figure 1:**
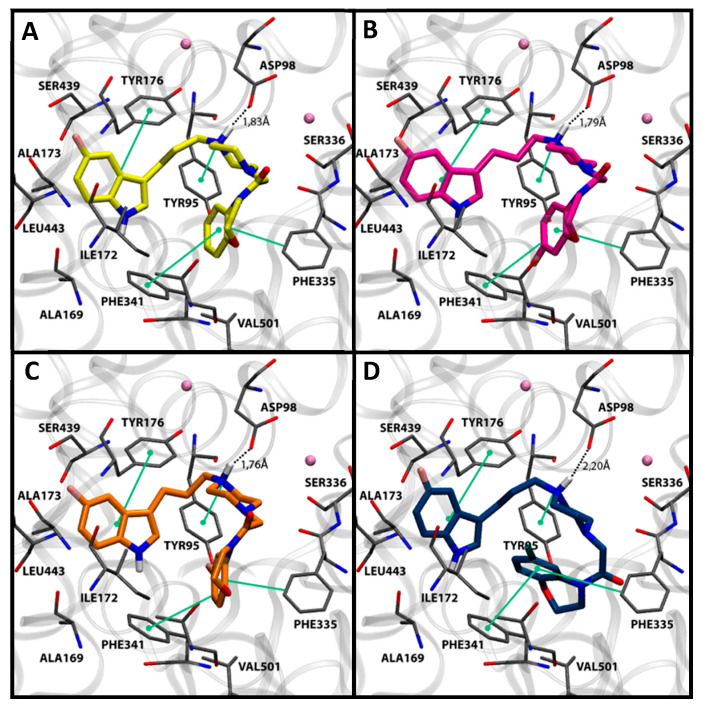
Docking poses in SERT obtained for compounds **7g** (**A**) in yellow, **7h** (**B**) in magenta, **7i** (**C**) in orange, and **7k** (**D**) in blue. Nearby residues <5 Å (grey sticks) and Na^+^ atoms (pink spheres) are shown. Dotted lines represent ionic interactions and aromatic interactions are shown with green lines.

The most potent compounds **7g**, **7h**, **7i**, and 7**k** showed a common docking pose (Figure 1), which favors the following stabilizing interactions: A π–π interaction between the indole ring and the π–donor aromatic residue Tyr176, a coulombic interaction between the protonated piperazine N-1 with the Asp98 residue, and a π–cation interaction for the protonated piperazine with Tyr95. Furthermore, aromatic interactions were also observed for the benzoxazine ring with the residues Phe341 and Phe335. These drug–target interactions are in agreement with those described in the crystal structure of the hSERT in complex with the inhibitor (*S*)-citalopram [47,48].

**Figure 2:**
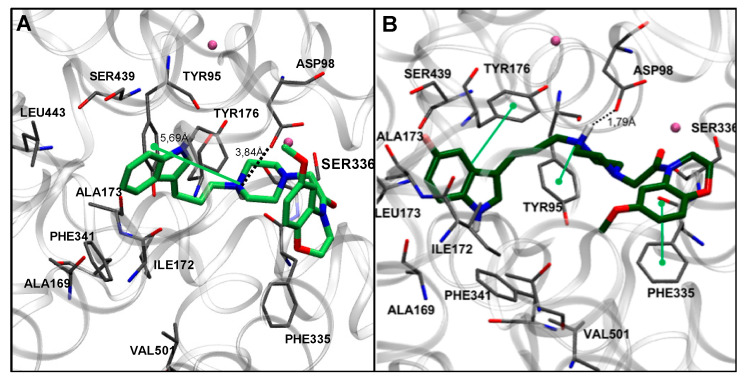
Comparison of the docking poses in SERT obtained for compounds **7f** (**A**) in light green and **7l** (**B**) in dark green. Nearby residues <5 Å (grey sticks) and Na^+^ atoms (pink spheres) are shown. Dotted lines represent ionic interactions and aromatic interactions are shown with green lines.

The relevance of the fluorinated substitution on the indole ring is clearly evidenced by comparison of compounds **7f** and **7l**. Both derivatives share the same substitution pattern in the benzoxazine ring, differing only by the presence of a fluorine atom at the indole moiety, which induces a different docking pose for **7f**. Thus, the least potent compound of the series (**7f**) adopted a binding mode in which both indole and piperazine ring interactions are clearly less favored than the C-5 fluorinated counterpart (Figure 2). Compounds showing intermediate affinities (**7a**–**7e** and **7m**–**7o**) exhibited docking poses between the most and least favorable binding modes (not shown).

#### 2.4.2. D_2_ Receptor

**Figure 3:**
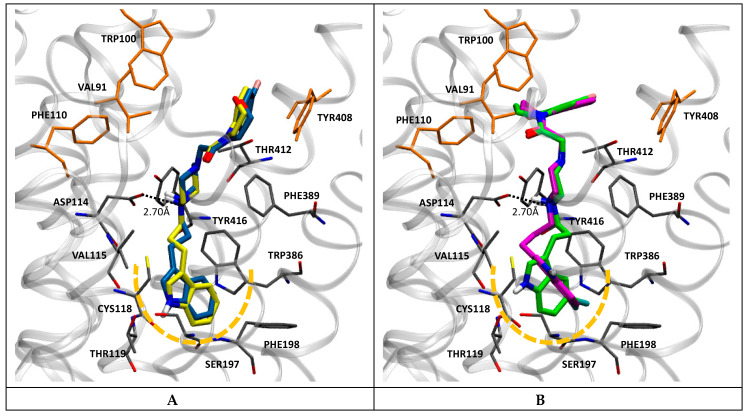
Docking poses in D_2_ obtained for compounds **7a** in yellow and **7b** in blue (**A**), and compounds **7m** in magenta and **7n** in green (**B**). Nearby residues < 5 Å (grey sticks) are shown. Dotted lines represent ionic interactions. Yellow segmented line represents the deep hydrophobic sub-pocket. Residues lining the more external hydrophobic sub-pocket are shown in orange.

Docking simulations showed that compounds of this series adopt, at the D_2_ receptor, a binding mode similar to that experimentally determined for the atypical antipsychotic risperidone [49]. Thus, the indole moiety appears located into the deep hydrophobic sub-pocket of the orthosteric site, lined by Cys118, Thr119, Ser197, Phe198, and Trp386, while the protonated piperazine N-1 locates in a favorable position to establish a coulombic interaction with Asp114 (Figure 3). Furthermore, the benzoxazine portion extends to the additional hydrophobic sub-pocket lined by Val91, Trp100, Phe110, and Tyr408, in a similar fashion to that observed in the crystal structure for the pyrimidinone moiety of risperidone. Interestingly, this general binding mode was observed for both the most and the least potent compounds of this series (**7a**, **7b**, and **7m**, **7n**), respectively (Figure 3A,B). Therefore, it is tempting to speculate that the higher affinity showed by brominated derivatives (**7m** and **7n**) is due to the formation of a halogen bond between the bromine and a hydroxyl group of an adjacent residue (e.g., Ser197). As observed (Figure 3), this could also change the position of the benzoxazine moiety, favoring its interactions at the more external hydrophobic sub-pocket.

### 2.5. Pharmacology Series II

Table 2 summarizes the affinity of Series II compounds **13a**–**13l** for SERT, D_2_ receptor, and MAO-A. As in the case of indole benzoxazine derivatives (Series I), most indole morpholine ethylbenzamides (Series II) were potent SERT ligands, showing much lower affinities for D_2_ receptor and virtually no effect upon MAO-A activity. Regarding SERT activity, and in agreement with our previous studies, halogen substitution at C-5 of the indole ring with fluorine or bromine (compounds **13a**–**g**) conducted an increase in affinity as compared with the unsubstituted analogues **13i**–**l**, with the fluoro derivatives **13a**–**d** being the most potent of the series. On the other hand, when the acetanilide portion, connected to the indolylpropylpiperazinyl fragment, was functionalized with a fluorine atom (at C-2) and a morpholino ethylcarboxamide, the best affinities were obtained when the bulkier substituent was located at meta position (compounds **13c**, **13g**, and **13k**).

### 2.6. Docking Simulation Series II

**Figure 4:**
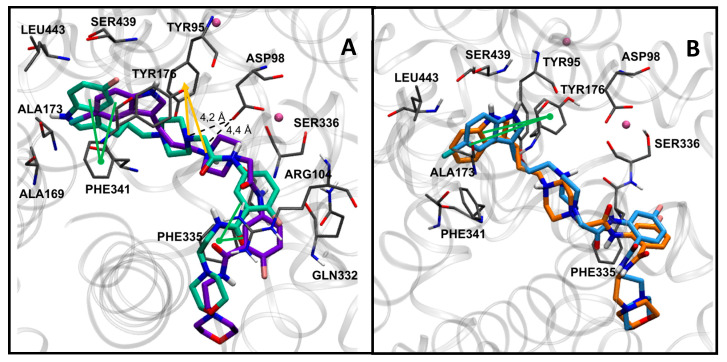
Docking poses in SERT obtained for compounds **13c** in cyan and **13g** in purple (**A**), and **13e** in light blue, and **13i** in orange (**B**). Nearby residues < 5 Å (grey sticks) and Na^+^ atoms (pink spheres) are shown. Dotted lines represent ionic interactions, orange lines represent π–cation interactions, and aromatic interactions are shown with green lines.

Similar to the analysis of Series I and considering the pharmacological results, docking studies were carried out only in hSERT. In this series, seven compounds exhibited Ki values between 7 and 60 nM (**13a**, **13b**, **13c**, **13f**, **13g**, **13k**, and **13l**). Docking simulations showed that compounds with the lowest Ki values (**13c** and **13g**) share a common binding mode into the S1 site of the SERT, which is similar to that described for compounds of Series I (Figure 4A). Thus, the piperazine N-1 can establish ionic and π–cation interactions with Asp98 and Tyr176, respectively, while the indole moiety can participate in aromatic interactions with Tyr176 and Phe341. Interestingly, the ethylmorpholinic chain extends towards the extracellular vestibule (also known as the S2 site). On the other hand, for the compounds with the lowest affinities (**13e** and **13i**), docking simulations showed that the piperazine N-1 was located farther away from Asp98 and Tyr95, making the possible ionic interactions with these residues unlikely or much weaker (Figure 4B). The analysis of the docking poses indicates that the most potent compounds, i.e., those having a 5,2-substitution pattern (**13c**, **13g**, and **13k**) exhibited an extended conformation at the binding site, while the least potent compounds (**13e** and **13i**, showing a 2,4-substitution pattern) adopted a more constrained binding mode, impairing the most relevant interactions.

### 2.7. 3-D-QSAR Study

To systematize the structure–activity relationship of the synthesized molecules, we carried out a 3-D-QSAR study of the CoMFA and CoMSIA type. The complete series of 27 molecules was divided into training (19 compounds) and test sets (8 compounds) in a ratio of 70:30, selecting the test set compounds at random to avoid bias. The q^2^ values for the best models were 0.625 and 0.523 for CoMFA and CoMSIA, respectively while the r^2^_ncv_ values were 0.967 and 0.959 for CoMFA and CoMSIA, respectively. The statistical summary, as well as the tables of affinities for both models and their respective graphs, are incorporated in the Supplementary Material.

**Figure 5:**
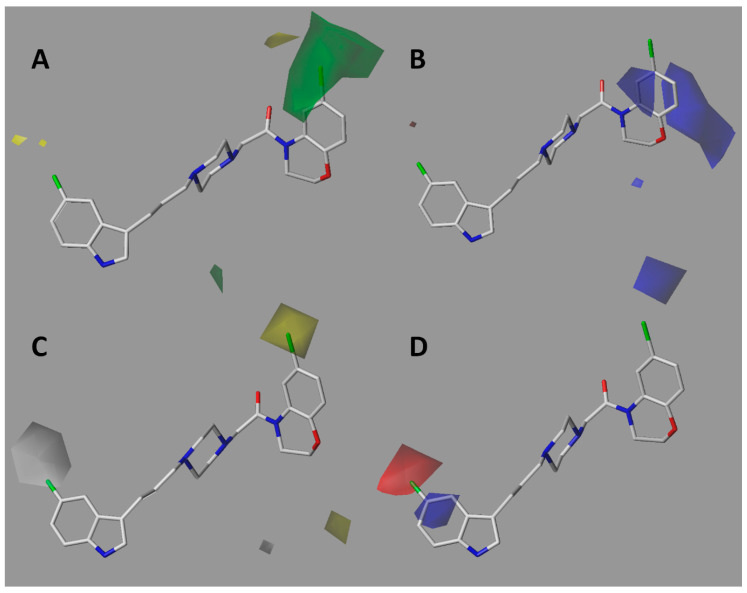
CoMFA (**A**,**B**) and CoMSIA (**C**,**D**) contour maps for the entire series of compounds evaluated in SERT. The compound with the best SERT affinity (**7k**) is depicted. A. Steric contour map. Green means that large groups are favorable for activity. B and D. Electrostatic contour maps. Red means electron-rich atoms are favorable and blue means positively charged atoms are favorable. C. Hydrophobic map. Gray means that hydrophilic groups are favorable for activity and yellow means that hydrophobic groups are favorable.

The steric contour map of CoMFA (Figure 5A) shows a green polyhedron on the bromine atom of compound **7k**, the most active of the series. This means that the insertion of bulky atoms or groups in this position is favorable for biological activity. This is consistent with docking studies showing that compounds of series I place halogen into the void space close to lipophilic residues like Trp100 and Tyr408. In the case of compounds of series II, the meta-substituted benzamides placed the chain towards the green region, not the ortho-substituted ones, so it is preferable that the chains are in the meta-position. This is confirmed in the docking of these compounds, in which better accommodation is observed in the SERT binding site. On the other hand, the electrostatic contour map (Figure 5B) shows three blue polyhedra of significant size. This means that the presence of positively charged atoms in these positions would be favorable for affinity. Such polyhedra are located on the carbon atom bonded to the halogen in the case of series I, suggesting that the presence of electronegative atoms bonded to the aforementioned carbon is favorable. The second blue polyhedron is localized on the oxygen atom of the carbonyl group belonging to the ortho-substituted series II amide-compounds. Therefore, oxygen atom remotion would be favorable for affinity. Finally, the third polyhedron is observed on the oxygen atom of the morpholine ring in the ortho-substituted compounds for series II, indicating that changing the morpholine by a piperazine or piperidine ring should lead to better affinities. Furthermore, alkyl chains substitutions at the ortho-position in the benzamide ring resulted in less favorable affinities compared to meta substitutions as was experimentally corroborated.

The hydrophobic contour map of CoMSIA (Figure 5C) showed a gray polyhedron at position C5 of the indole ring, meaning that the presence of hydrophilic groups is favorable for affinity. In fact, the C5 fluorine-substituted indoles displayed the best affinities of the series. Other polar groups like OH, NH_2_, or NR_2_ would also be interesting to evaluate at this position. Similarly, a yellow polyhedron located on the bromine atom of the benzoxazine framework (compound **7k**) means that the presence of lipophilic groups is favorable for activity. In concordance, halogens like Cl, Br, and I would be the most appropriated substituents and groups, such as aromatic rings, alkyl, and/or alkoxy chains, could also be explored. In the case of compounds of series II, a yellow polyhedron is located on the amide group of the meta-substituted compounds; therefore, the replacement of the amide by a less-polar function, such as a ketone or ester, would be an interesting option to explore. On the other hand, the electrostatic contour map of CoMSIA (Figure 5D) showed two polyhedra around compound **7k**. A red polyhedron on the halogen atom at position 5 of the indole ring means that the presence of electron-rich atoms is favorable for affinity. It is interesting to note that the blue polyhedron intersecting the carbon atom of indole at position 5 is complementary to the red polyhedron. In consequence, the presence of a positive charge on the indole ring is favorable for activity. Other potential electron-withdrawing groups to be explored are CN, NO_2_, and COR. Docking studies showed π–stacking interaction between the π-deficient indole ring with Tyr176, Phe341, and Trp386 residues.

## 3. Materials and Methods

### 3.1. General Methods

Melting points were determined on a hot-stage apparatus and were uncorrected. The ^1^H and ^13^C-NMR spectra were obtained on a Bruker DRX-300 spectrometer (300 and 75 MHz, respectively) in CDCl_3_, DMSO-*d*_6_, and CD_3_COCD_3_-*d*_6_. Chemical shifts were recorded in ppm (δ) relative to TMS as an internal standard. *J* values are given in Hz. Micro-analyses were carried out on a Fisons EA 1108 analyzer. High-resolution mass spectra were recorded on a DSA–TOFAxION 2 TOF MS (Perkin Elmer, Shelton, CT, USA), positive mode. Silica gel Merck 60 (70–230 mesh) and aluminum sheets coated with silica gel 60 F254 were used for column and TLC chromatography, respectively.

#### 3.1.1. General Procedure for the Synthesis of [4-[2-(3,4-Dihydro-2*H*-benzo[*b*][1,4]oxazin-4-yl)-2-oxo-ethyl]-piperazine-1-yl] tert-butylcarbamate Derivatives **5a**–**c**

##### [4-[2-(3,4-Dihydro-2*H*-benzo[*b*][1,4]oxazin-4-yl)-2-oxo-ethyl]-1-piperazinyl] tert-butylcarbamate (**5a**) as a Model

To a solution containing 2-Chloro-1-(2,3-dihydrobenzo[*b*][1,4]oxazin-4-yl) ethanamide **4a** (1.5 g; 7.09 mmol) in dry CH_3_CN (60 mL), *N*-Boc-piperazine (1321 mg; 7.09 mmol) and anhydrous K_2_CO_3_ (980 mg; 7.09 mmol) were added. The mixture was stirred at 80 °C for 24 h. After this time, the mixture was diluted with water (100 mL) and the solution extracted with EtOAc (100 mL × 3), dried over anhydrous Na_2_SO_4_, and concentrated under reduced pressure. The organic crude was purified by silica gel column chromatography with EtOAc as eluent, to provide **5a** (2152 mg; 84% yield) as a white solid. m.p.: 133.0–134.0 °C; ^1^H-NMR (CDCl_3_): δ 1.45 (s, 9H, H-4′), 2.52 (t, 4H, H-3′,*J* = 4.8 Hz), 3.39 (s, 2H, H-1′), 3.45 (t, 4H, H-2′, *J* = 4.5 Hz), 3.99 (t, 2H, H-3, *J* = 4.8 Hz), 4.31 (t, 2H, H-2, *J* = 4.4 Hz), 6.91 (m, 2H, H-6 and H-8), 7.08 (t, 1H, H-7, *J* = 7.3 Hz), and 7.99 (br. s, 1H, H-5) ppm. ^13^C-NMR (CDCl_3_): δ 27.9 (3X), 42.6, 43.2 (2X), 52.4 (2X), 55.4, 66.4, 79.3, 116.8, 119.7, 123.5, 125.6, 146.3, 154.2, 167.3, and 167.5 ppm. HRMS: (EI) Calculated for C_19_H_27_N_3_O_4_ (M^+^) = 362.20799. Found: 362.2113.

##### [4-[2-(7-Fluoro-2,3-dihydro-benzo[*b*][1,4]oxazin-4-yl)-2-oxo-ethyl]-1-piperazinyl] tert-butylcarbamate (**5b**)

2-Chloro-1-(7-fluoro-2,3-dihidro-benzo [1,4]oxazin-4-yl)-ethanamide **4b** (1.5 g; 6.53 mmol), *N*-Boc-piperazine (1216 mg; 6.53 mmol), and anhydrous K_2_CO_3_ (902 mg; 6.53 mmol), to afford **5b** (1983 mg; 80% yield) as a white solid. m.p.: 126.6–128.5 °C; ^1^H-NMR (CDCl_3_): δ 1.46 (s, 9H, H-4′), 2.51 (t, 4H, H-2′, *J* = 4.9 Hz), 3.35 (s, 2H, H-1′), 3.45 (t, 4H, H-3′, *J* = 4.8 Hz), 3.97 (t, 2H, H-3, *J* = 4.8 Hz), 4.30 (t, 2H, H-2, *J* = 4.1 Hz), 6.58–6.66 (m, 2H, H-6, and H-8), and 8.03 (br. s, 1H, H-5) ppm. ^13^C-NMR (CDCl_3_): δ 27.9 (3X), 42.5 43.2 (2X), 52.4 (2X), 62.1, 66.4, 79.3, 103.8 (d, ^2^*J*_C-F_ = 27 Hz), 106.7 (d, ^2′^*J*_C-F_ = 22.5 Hz), 121.8 (d, ^3^*J*_C-F_ = 9.3 Hz), 124.6 (d, ^4^*J*_C-F_ = 8.2 Hz), 147.3 (d, ^3′^*J*_C-F_ = 6 Hz), 154.2, 155.6 (d, ^1^*J*_C-F_ = 222 Hz), and 167 ppm. HRMS: (EI) Calculated for C_19_H_26_FN_3_O_4_ (M^+^) = 380.19856. Found: 380.2047.

##### [4-[2-(6-Fluoro-2,3-dihydro-benzo[*b*][1,4]oxazin-4-yl)-2-oxo-ethyl]-1-piperazinyl] tert-butylcarbamate (**5c**)

2-Chloro-1-(6-fluoro-2,3-dihidro-benzo [1,4]oxazin-4-yl)-ethanamide **4c** (1.5 g; 6.53 mmol), *N*-Boc-piperazine (1216 mg; 6.53 mmol), and anhydrous K_2_CO_3_ (902 mg; 6.53 mmol), to afford **5c** (2033 mg; 82% yield) as a white solid. m.p.: 113.4–114.5 °C; ^1^H-NMR (CDCl_3_): δ 1.46 (s, 9H, H-4′), 2.53 (t, 4H, H-2′, *J* = 4.8 Hz), 3.35 (s, 2H, H-1′), 3.46 (t, 4H, H-3′, *J* = 4.8 Hz), 3.98 (t, 2H, H-3, *J* = 4.9 Hz), 4.27 (t, 2H, H-2, *J* = 4.4 Hz), 6.77–6.87 (m, 2H, H-6 and H-8), and 7.82 (br. s, 1H, H-5) ppm. ^13^C-NMR (CDCl_3_): δ 27.9 (3X), 42.5 43.4 (2X), 52.3 (2X), 62.1, 65.6, 79.3, 110.1 (d, ^2^*J*_C-F_ = 28.5 Hz), 112.2 (d, ^2′^*J*_C-F_ = 23.6 Hz), 117.1 (d, ^3^*J*_C-F_ = 9.3 Hz), 125.7 (d, ^4^*J*_C-F_ = 4.9 Hz), 142.2 (d, ^3′^*J*_C-F_ = 9.3 Hz),154.2, 155.5 (d, ^1^*J*_C-F_ = 238 Hz), and 167.1 ppm. HRMS: (EI) Calculated for C_19_H_26_FN_3_O_4_ (M^+^) = 380.19856. Found: 380.2043.

#### 3.1.2. General Procedure for the Synthesis of 1-(2,3-dihydro-benzo[1,4]oxazin-4-yl)-2-piperazin-1-yl-ethanamide Derivatives **6a**–**c**

##### Synthesis of 1-(2,3-Dihydro-benzo[*b*][1,4]oxazin-4-yl)-2-(1-piperazinyl) ethanamide (**6a**) as a Model

A mixture of [4-[2-(3,4-Dihydro-2*H*-benzo[*b*][1,4]oxazin-4-yl)-2-oxo-ethyl]-1-piperazinyl] tert-butylcarbamate **5a** (2 g; 5.53 mmol) in dry CH_2_Cl_2_ (20 mL) and trifluoroacetic acid (12 mL) was stirred at 0 °C, for 4 h. After this time, dry CH_2_Cl_2_ (200 mL) was added and neutralized with solid NaHCO_3_ (10 g) to later filter on celite. The mixture was finally diluted with a saturated solution of NaHCO_3_ (200 mL), extracted with EtOAc (8 × 50 mL), dried over anhydrous Na_2_SO_4_, and concentrated under vacuum to obtain pure **6a** (867 mg; 82% yield) as an unstable yellow light solid, highly hygroscopic; ^1^H-NMR (DMSO-*d*_6_): δ 2.37 (m, 4H, H-2′), 2.69 (m, 4H, H-3′), 3.29 (s, 2H, H-1′), 3.89 (t, 2H, H-3, *J* = 4.4 Hz), 4.25 (t, 2H, H-2, *J* = 4.1 Hz), 6.85 (m, 2H, H-6 and H-8), 7.0 (t, 1H, H-7, *J* = 7.6 Hz) and 7.85 (br. s, 1H, H-5) ppm. ^13^C-NMR (DMSO-*d*_6_): δ40.5, 45.2 (2X), 53.2 (2X), 62.0, 66.1, 73.5, 116.7, 119.6, 123.8, 125.1, 146.2, and 167.8 ppm.

##### 1-(7-Fluoro-2,3-dihydro-benzo[*b*][1,4]oxazin-4-yl)-2-(1-piperazinyl) ethanamide (**6b**)

[4-[2-(7-Fluoro-2,3-dihydro-benzo[*b*][1,4]oxazin-4-yl)-2-oxo-ethyl]-1-piperazinyl]tertbutyl carbamate **5b** (2 g; 5.27 mmol) and trifluoroacetic acid (12 mL), in dry CH_2_Cl_2_ (20 mL) reacted to provide **6b** (1398 mg; 95% yield) as a brown light hygroscopic compound; ^1^H-NMR (CDCl_3_): δ 2.5 7 (m, 4H, H-2′), 2.94 (t, 4H, H-3′, *J* = 4.4 Hz), 3.18 (br. s, 1H, H-4′), 3.33 (s, 2H, H-1′), 3.97 (t, 2H, H-3, *J* = 4.8 Hz), 4.31 (t, 2H, H-2, *J* = 4.2 Hz), 6.59–6.65 (m, 2H, H-6, and H-8), and 8.0 (br. s, 1H, H-5) ppm. ^13^C-NMR (CDCl_3_): δ 45.6 (2X), 53.8 (2X), 60.4, 66.9, 77.3, 104.2 (d, ^2^*J*_C-F_ = 24 Hz), 107.2 (d, ^2′^*J*_C-F_ = 23 Hz), 114.5 (d, ^4^*J*_C-F_ = 7 Hz), 125.1 (d, ^3^*J*_C-F_ = 9.4 Hz), 143.4 (d, ^3′^*J*_C-F_ = 15 Hz), 155.7 (d, ^1^*J*_C-F_ = 250 Hz), and 171.2 ppm.

##### 1-(6-Fluoro-2,3-dihydro-benzo[*b*][1,4]oxazin-4-yl)-2-(1-piperazinyl) ethanamide (**6c**)

[4-[2-(6-Fluoro-2,3-dihydro-benzo[*b*][1,4]oxazin-4-yl)-2-oxo-ethyl]-1-piperazinyl]tertbutyl carbamate **5c** (2 g; 5.27 mmol) and trifluoroacetic acid (12 mL), in dry CH_2_Cl_2_ (20 mL) reacted to provide **6c** (1369 mg; 93% yield) as a brown light hygroscopic compound; ^1^H-NMR (CDCl_3_): δ 2.62 (m, 4H, H-2′), 2.99 (t, 4H, H-3′, *J* = 4.6 Hz), 3.35 (s, 2H, H-1′), 3.97 (m, 3H, H-3 and H-4′), 4.28 (t, 2H, H-2, *J* = 4.5 Hz), 6.76–6.87 (m, 2H, H-7 and H-8) and 7.71 (br. s, 1H, H-5) ppm; ^13^C-NMR (CDCl_3_): δ 45.6 (2X), 53.8 (2X), 60.4, 66.9, 77.2, 110.6 (d, ^2^*J*_C-F_ = 28 Hz), 112.8 (d, ^2′^*J*_C-F_ = 32 Hz), 117.6 (d, ^3^*J*_C-F_ = 9.4 Hz), 126.1 (d, ^3′^*J*_C-F_ = 11 Hz), 142.8 (d, ^4^*J*_C-F_ = 6.6 Hz), 157.6(d, ^1^*J*_C-F_ = 237 Hz), and 171.2 ppm.

#### 3.1.3. General Procedure for the Synthesis of 2,3-dihydro-benzo[*b*][1,4] oxazin-4-yl)-2-{4-[3-(1*H*-3-indolyl)-propyl]-1-piperazinyl} ethanamide Derivatives **7a**–**c**, **7g–i**, and **7m–o**. Method 1

##### 1-(2,3-Dihydro-benzo[*b*][1,4]oxazin-4-yl)-2-{4-[3-(1*H*-3-indolyl)-propyl]-1-piperazinyl} ethanamide (**7a**) as a Model

To a stirred solution of 3-(1*H*-3-indolyl)-propyl-4-methylbencensulfonate **1a** (254 mg. 0.77 mmol), in dry CH_3_CN (50 mL), benzoxazine **6a** (200 mg, 0.77 mmol) and anhydrous K_2_CO_3_ (106 mg; 0.77 mmol) were added. The mixture was heated at 80 °C for 24 h. After this time, the resulting mixture was poured into water (100 mL), extracted with EtOAc (4 × 50 mL), dried over anhydrous Na_2_SO_4_, and concentrated under reduced pressure. The organic crude was purified by column chromatography EtOAc/MeOH (6:1) to give compound **7a** (217 mg; 70% yield) as a yellow light solid; m.p.: 124.3–125.8 °C; ^1^H-NMR (DMSO-*d*_6_): δ 1.77 (q, 2H, H-2′, *J* = 7.0 Hz), 2.22–2.50 (m, 10H, H-3′, H-4′ and H-5′), 2.67 (t, 2H, H-1′, *J* = 7.4 Hz), 3.33 (s, 2H, H-7′), 3.90 (t, 2H, H-3″, *J* = 3.8 Hz), 4.26 (t, 2H, H-2″, *J* = 3.7 Hz), 6.85 (m, 2H, H-6″ and H-8″), 6.95 (t, 1H, H-7″, *J* = 7.7 Hz), 7.03 (m, 2H, H-5 and H-6), 7.09 (d, 1H, H-2, *J* = 1.4 Hz), 7.33 (d, 1H, H-4, *J* = 7.8 Hz), 7.49 (d, 1H, H-7, *J* = 7.7 Hz), 7.90 (br. s, 1H, H-5″), and 10.79 (br. s, 1H, H-1) ppm. ^13^C-NMR (DMSO-*d*_6_): δ 22.9, 27.6, 39.2, 40.8, 53.0 (2X), 53.2 (2X), 58.0, 66.6, 111.8, 114.8, 117.2, 118.5, 118.7, 120.1, 121.2, 122.6, 124.3, 126.0, 127.7, 128.5, 136.7, 146.7, and 168.3 ppm. HRMS: (EI) Calculated for C_25_H_30_N_4_O_2_ (M^+^) = 419.5313. Found: 419.4426.

##### 1-(7-Fluoro-2,3-dihydro-benzo[*b*][1,4]oxazin-4-yl)-2-{4-[3-(1*H*-3-indolyl)-propyl]-1-piperazinyl} ethanamide (**7b**)

To a solution of 3-(1*H*-3-indolyl)-propyl-4-methylbencensulfonate **1a** (237 mg; 0.72 mmol), in dry CH_3_CN (50 mL), 7-fluoro-benzoxazine **6b** (200 mg; 0.72 mmol) and anhydrous K_2_CO_3_ (100 mg; 0.72 mmol) were added. The chromatographic purification provided **7b** (242 mg; 80% yield) as a yellow light solid. m.p.: 124.0–125.3 °C; ^1^H-NMR (DMSO-*d*_6_): δ 1.76 (q, 2H, H-2′, *J* = 7.3 Hz), 2.22–2.48 (m, 10H, H-3′, H-4′, and H-5′), 2.67 (t, 2H, H-1′, *J* = 7.4 Hz), 3.31 (s, 2H, H-7′), 3.90 (m, 2H, H-3″), 4.29 (m, 2H, H-2″), 6.68–6.78 (m, 2H, H-6″ and H-8″), 6.95 (t, 1H, H-5 or H-6, *J* = 7.1 Hz), 7.04 (t, 1H, H-6 or H-5, *J* = 7.0 Hz), 7.09 (d, 1H, H-2, *J* = 1.8 Hz), 7.32 (d, 1H, H-4, *J* = 8.0 Hz), 7.49 (d, 1H, H-7, *J* = 7.7 Hz), 7.92 (br. s, 1H, H-5″), and 10.83 (br. s, 1H, H-1) ppm. ^13^C-NMR (DMSO-*d*_6_): δ 22.9, 27.6, 53.0 (2X), 53.3 (2X), 58.1, 67, 104.0 (d, ^2^*J*_C-F_ = 26 Hz), 106.9 (d, ^2′^*J*_C-F_ = 23 Hz), 111.8, 114.8, 118.6 (d, ^3^*J*_C-F_ = 18 Hz), 121.2, 122.6, 123.2, 125.6 (d, ^4^*J*_C-F_ = 9 Hz), 127.7, 136.7, 143.2, 144, 147.6, 147.8 (d, ^3′^*J*_C-F_ = 13 Hz), 159.5 (d, ^1^*J*_C-F_ = 240 Hz), and 168.2 ppm. HRMS: (EI) Calculated for C_25_H_29_FN_4_O_2_ (M^+^) = 437.5318. Found: 437.5301.

##### 1-(6-Fluoro-2,3-dihydro-benzo[*b*][1,4]oxazin-4-yl)-2-{4-[3-(1*H*-3-indolyl)-propyl]-1-piperazinyl} ethanamide (**7c**)

To a solution of 3-(1*H*-3-indolyl)-propyl-4-methylbencensulfonate **1a** (237 mg; 0.72 mmol), in dry CH_3_CN (50 mL), 6-fluoro-benzoxazine **6c** (200 mg; 0.72 mmol) and anhydrous K_2_CO_3_ (100 mg; 0.72 mmol) were added. The crude mixture was column chromatographed to provide pure **7c** (269 mg; 89% yield) as a yellow light solid. m.p.: 123.8–125.1 °C; ^1^H-NMR (DMSO-*d*_6_): δ 1.83 (q, 2H, H-2′, *J* = 7.1 Hz), 2.30–2.54 (m, 10H, H-3′, H-4′, and H-5′), 2.73 (t, 2H, H-1′, *J* = 7.4 Hz), 3.40 (s, 2H, H-7′), 3.98 (t, 2H, H-3″, *J* = 4.0 Hz), 4.30 (t, 2H, H-2″, *J* = 3.7 Hz), 6.91–6.97 (m, 2H, H-7″ and H-8″), 7.01 (td, 1H, H-5 or H-6, *J_o_* = 7.3 Hz, *J_m_* = 1.0 Hz), 7.11 (td, 1H, H-6 or H-5, *J_o_* = 7.0 Hz, *J_m_* = 1.1 Hz), 7.15 (d, 1H, H-2, *J* = 2.1 Hz), 7.38 (d, 1H, H-4, *J* = 8.0 Hz), 7.55 (d, 1H, H-7, *J* = 7.7 Hz), 7.95 (br. d, 1H, H-5″, *J* = 11.7 Hz), and 10.81 (br. s, 1H, H-1) ppm. ^13^C-NMR (DMSO-*d*_6_): δ 22.9, 27.6, 53.0 (2X), 53.3 (2X), 58.1, 66.1, 110.3 (d, ^2^*J*_C-F_ = 29 Hz), 111.9 (d, ^2′^*J*_C-F_ = 25 Hz), 114.8, 117.9 (d, ^3^*J*_C-F_ = 9 Hz), 118.5, 118.7, 121.2, 122.6, 126.9 (d, ^3′^*J*_C-F_ = 12 Hz), 127.7, 128.7, 129.9, 136.7, 138.3, 143 (d, ^4^*J*_C-F_ = 2 Hz), 155.5 (d, ^1^*J*_C-F_ = 239 Hz), and 168.7 ppm. HRMS: (EI) Calculated for C_25_H_29_FN_4_O_2_ (M^+^) = 437.5318. Found: 437.5084.

##### 1-(2,3-Dihydro-benzo[*b*][1,4]oxazin-4-yl)-2-{4-[3-(5-fluoro-1*H*-3-indolyl)-propyl]-1-piperazinyl} ethanamide (**7g**)

To a solution of 3-(5-Fluoro-1*H*-3-indolyl)-propyl-4-methylbencensulfonate **1b** (268 mg; 0.77 mmol) in CH_3_CN (50 mL), 1-(2,3-Dihydro-benzo[*b*][1,4]oxazin-4-yl)-2-(1-piperazinyl) ethanamide **6a** (200 mg; 0.77 mmol) and anhydrous K_2_CO_3_ (106 mg; 0.77 mmol) were added. The crude mixture was column chromatographed to provide pure **7g** (252 mg; 78% yield) as a yellow light solid. m.p.: 168.4–171.3 °C; ^1^H-NMR (DMSO-*d*_6_): δ 1.76 (q, 2H, H-2′, *J* = 6.9 Hz), 2.30–2.51 (m, 10H, H-3′, H-4′ and H-5′), 2.63 (t, 2H, H-1′, *J* = 7.3 Hz), 3.34 (s, 2H, H-7′), 3.89 (m, 2H, H-3″), 4.26 (t, 2H, H-2″, *J* = 3.6 Hz), 6.75 (td, 1H, H-6, *J_o_* = 10.4 Hz, *J_m_* = 2.7 Hz), 6.80–6.91 (m, 2H, H-6″, and H-8″), 7.01 (t, 1H, H-7″, *J* = 7.4 Hz), 7.18 (d, 1H, H-2, *J* = 1.8 Hz), 7.23 (dd, 1H, H-7, *J_o_* = 10.2 Hz, *J_m_* = 2.3 Hz), 7.30 (dd, 1H, H-4, *J_o_* = 8.9 Hz, *J_m_* = 4.5 Hz), 7.88 (br. s, 1H, H-5″), and 10.89 (s, 1H, H-1) ppm. ^13^C-NMR (DMSO-*d*_6_): δ 22.6, 27.2, 52.7 (2X), 53.1 (2X), 57.7, 66.1, 103.4 (d, ^2^*J*_C-F_ = 23 Hz), 104.2, 109.3 (d, ^2′^*J*_C-F_ = 26 Hz),112.6 (d, ^3^*J*_C-F_ = 9.9 Hz), 115.1 (d, ^4^*J*_C-F_ = 4.9 Hz), 117.2, 120.1, 124.3, 124.9, 125.7, 126.6, 127.9 (d, ^3′^*J*_C–F_ = 9.9 Hz), 133.4, 146.7, 147.9, 157.3 (d, ^1^*J*_C-F_ = 234 Hz), and 168.2 ppm. HRMS: (EI) Calculated for C_25_H_29_FN_4_O_2_ (M^+^) = 437.5318. Found: 437.5312.

##### 1-(7-Fluoro-2,3-dihydro-benzo[*b*][1,4]oxazin-4-yl)-2-{4-[3-(5-fluoro-1*H*-3-indolyl)-propyl]-1-piperazinyl} ethanamide (**7h**)

To a solution of 3-(5-Fluoro-1*H*-3-indolyl)-propyl-4-methylbencensulfonate **1b** (268 mg; 0.72 mmol) in CH_3_CN (50 mL), 1-(7-Fluoro-2,3-dihydro-benzo[*b*][1,4]oxazin-4-yl)-2-(1-piperazinyl) ethanamide **6b** (200 mg; 0.72 mmol) and anhydrous K_2_CO_3_ (100 mg; 0.72 mmol) were added. The crude mixture was column chromatographed to provide pure **7h** (205 mg; 65% yield) as a yellow light solid. m.p.: 168.1–169.9 °C; ^1^H-NMR (DMSO-*d*_6_): δ 1.75 (q, 2H, H-2′, *J* = 7.0 Hz), 2.25–2.52 (m, 10H, H-3′, H-4′ and H-5′), 2.64 (t, 2H, H-1′, *J* = 7.4 Hz), 3.35 (s, 2H, H-7′), 3.92 (t, 2H, H-3″, *J* = 3.8 Hz), 4.25 (t, 2H, H-2″, *J* = 3.7 Hz), 6.85–6.93 (m, 3H, H-6, H-6″, and H-8″), 7.19 (d, 1H, H-2, *J* = 2.1 Hz), 7.24 (dd, 1H, H-7, *J_o_* = 10.2 Hz, *J_m_* = 2.3 Hz), 7.31 (dd, 1H, H-4, *J_o_* = 8.8 Hz, *J_m_* = 4.5 Hz), 7.92 (br. d, 1H, H-5″, *J* = 9.6 Hz), and 10.88 (s, 1H, H-1) ppm. ^13^C-NMR (DMSO-*d*_6_): δ 22.7, 27.5, 52.9 (2X), 53.2 (2X), 57.8, 66.1, 103.4 (d, ^2^*J*_C-F_ = 23 Hz), 109.3 (d, ^2′^*J*_C-F_ = 26 Hz), 110.3 (d, ^2″^*J*_C-F_ = 28 Hz), 112.0 (d, ^2‴^*J*_C-F_ = 25 Hz), 112.6 (d, ^3^*J*_C-F_ = 9.9 Hz), 115.1 (d, ^4^*J*_C-F_ = 4.9 Hz), 117.9 (d, ^3′^*J*_C-F_ = 9 Hz), 124.9, 126, 126.9 (d, ^3″^*J*_C–F_ = 9 Hz), 127.9 (d, ^3‴^*J*_C–F_ = 9.9 Hz), 128.5, 133.3, 143.0 (d, ^4′^*J*_C-F_ = 2.3 Hz), 155.5 (d, ^1^*J*_C-F_ = 234 Hz), 157.0 (d, ^1′^*J*_C–F_ = 231 Hz), and 168.7 ppm. HRMS: (EI) Calculated for C_25_H_28_F_2_N_4_O_2_ (M^+^) = 455.5222. Found: 455.5198.

##### 1-(6-Fluoro-2,3-dihydro-benzo[*b*][1,4]oxazin-4-yl)-2-{4-[3-(5-fluoro-1*H*-3-indolyl)-propyl]-1-piperazinyl} ethanamide (**7i**)

To a solution of 3-(5-Fluoro-1*H*-3-indolyl)-propyl-4-methylbencensulfonate **1b** (268 mg; 0.72 mmol) in CH_3_CN (50 mL), 1-(6-Fluoro-2,3-dihydro-benzo[*b*][1,4]oxazin-4-yl)-2-(1-piperazinyl) ethanamide **6c** (200 mg; 0.72 mmol) and anhydrous K_2_CO_3_ (100 mg; 0.72 mmol) were added to afford pure **7i** (196 mg; 62% yield) as a yellow light solid. m.p.: 166.8–168.1 °C; ^1^H-NMR (DMSO-*d*_6_): δ 1.75 (q, 2H, H-2′, *J* = 7.0 Hz), 2.22–2.55 (m, 10H, H-3′, H-4′ and H-5′), 2.64 (t, 2H, H-1′, *J* = 7.4 Hz), 3.35 (s, 2H, H-7′), 3.92 (t, 2H, H-3″, *J* = 3.8 Hz), 4.25 (t, 2H, H-2″, *J* = 3.7 Hz), 6.83–6.94 (m, 3H, H-6, H-7″ and H-8″), 7.19 (d, 1H, H-2, *J* = 2.1 Hz), 7.24 (dd, 1H, H-7, *J_o_* = 10.2 Hz, *J_m_* = 2.3 Hz), 7.31 (dd, 1H, H-4, *J_o_* = 8.8 Hz, *J_m_* = 4.5 Hz), 7.92 (br. d, 1H, H-5″, *J* = 9.6 Hz), and 10.88 (s, 1H, H-1) ppm. ^13^C-NMR (DMSO-*d*_6_): δ 22.7, 27.5, 52.9 (2X), 53.2 (2X), 57.8, 66.1, 103.4 (d, ^2^*J*_C-F_ = 23 Hz), 109.3 (d, ^2′^*J*_C-F_ = 26 Hz), 110.3 (d, ^2″^*J*_C-F_ = 28 Hz), 112.0 (d, ^2‴^*J*_C-F_ = 24.7 Hz), 112.6 (d, ^3^*J*_C-F_ = 9.9 Hz), 115.1 (d, ^4^*J*_C-F_ = 4.9 Hz), 117.9 (d, ^3″^*J*_C-F_ = 8.8 Hz), 124.9, 126.9 (d, ^3′^*J*_C–F_ = 13 Hz), 126, 127.9 (d, ^3‴^*J*_C–F_ = 9.9 Hz), 129, 133.3, 143.0 (d, ^4′^*J*_C-F_ = 2.2 Hz), 155.5 (d, ^1^*J*_C-F_ = 234 Hz), 157.0 (d, ^1′^*J*_C–F_ = 231 Hz), and 168.6 ppm. HRMS: (EI) Calculated for C_25_H_28_F_2_N_4_O_2_ (M^+^) = 455.5222. Found: 455.5205.

##### 1-(2,3-Dihydro-benzo[*b*][1,4]oxazin-4-yl)-2-{4-[3-(5-bromo-1*H*-3-indolyl)-propyl]-1-piperazinyl} ethanamide (**7m**)

To a solution of 3-(5-Bromo-1*H*-3-indolyl)-propyl-4-methylbencensulfonate **1c** (314 mg; 0.77 mmol) in CH_3_CN (50 mL), 1-(2,3-Dihydro-benzo[*b*][1,4]oxazin-4-yl)-2-(1-piperazinyl) ethanamide **6a** (200 mg; 0.77 mmol) and anhydrous K_2_CO_3_ (106 mg; 0.77 mmol) were added. The crude mixture was column chromatographed to provide pure **7m** (157 mg; 42% yield) as a yellow light solid. m.p.: 90.5–92.4 °C; ^1^H-NMR (DMSO-*d*_6_): δ 1.74 (q, 2H, H-2′, *J* = 7.1 Hz), 2.25–2.51 (m, 10H, H-3′, H-4′, and H-5′), 2.65 (t, 2H, H-1′, *J* = 7.1 Hz), 3.34 (s, 2H, H-7′), 3.90 (t, 2H, H-3″, *J* = 7.1 Hz), 4.27 (t, 2H, H-2″, *J* = 7.1 Hz), 6.81–6.90 (m, 2H, H-6″, and H-8″), 7.02 (t, 1H, H-7″, *J* = 7.6 Hz), 7.10–7.20 (m, 2H, H-2, and H-6), 7.30 (d, 1H, H-7, *J* = 8.5 Hz), 7.68 (d, 1H, H-4, *J* = 1.6 Hz), 7.85 (br. s, 1H, H-5″), and 11.04 (s, 1H, H-1) ppm. ^13^C-NMR (DMSO-*d*_6_): δ 22.4, 27.6, 52.9 (2X), 53.2 (2X), 57.6, 66.6, 111.2, 113.9, 114.7, 117.2, 120.1, 121.1, 123.6, 124.3, 124.5, 125.7, 126, 126.6, 128.5, 129.6, 135.3, 146.7, and 168.2 ppm. HRMS: (EI) Calculated for C_25_H_29_BrN_4_O_2_ (M^+^) = 498.4374. Found: 498.4362.

##### 1-(7-Fluoro-2,3-dihydro-benzo[*b*][1,4]oxazin-4-yl)-2-{4-[3-(5-bromo-1*H*-3-indolyl)-propyl]-1-piperazinyl} ethanamide (**7n**)

To a solution of 3-(5-Bromo-1*H*-3-indolyl)-propyl-4-methylbencensulfonate **1c** (294 mg; 0.72 mmol) in CH_3_CN (50 mL), 1-(7-Fluoro-2,3-dihydro-benzo[*b*][1,4]oxazin-4-yl)-2-(1-piperazinyl) ethanamide **6b** (200 mg; 0.72 mmol) and anhydrous K_2_CO_3_ (100 mg; 0.72 mmol) were added. The crude mixture was column chromatographed to provide pure **7n** (180 mg; 50% yield) as a yellow light solid. m.p.: 96.1–98.1 °C; ^1^H-NMR (DMSO-*d*_6_): δ 1.73 (q, 2H, H-2′, *J* = 6.6 Hz), 2.18–2.51 (m, 10H, H-3′, H-4′and H-5′), 2.64 (t, 2H, H-1′, *J* = 7.0 Hz), 3.32 (s, 2H, H-7′), 3.90 (m, 2H, H-3″), 4.29 (m, 2H, H-2″), 6.65–6.81 (m, 2H, H-6″ and H-8″), 7.10–7.20 (m, 2H, H-2, and H-6), 7.30 (d, 1H, H-7, *J* = 8.5 Hz), 7.68 (d, 1H, H-4, *J* = 1.0 Hz), 7.95 (br. s, 1H, H-5″), and 11.05 (s, 1H, H-1) ppm. ^13^C-NMR (DMSO-*d*_6_): δ 22.4, 27.6, 52.9 (2X), 53.2 (2X), 57.6, 66.6, 110.3 (d, ^2^*J*_C-F_ = 28.5 Hz), 111.2, 112.0 (d, ^2′^*J*_C-F_ = 25 Hz),113.8, 114.7, 117.9 (d, ^3^*J*_C-F_ = 8.8 Hz), 121.1, 123.6, 124.4, 126.9 (d, ^3′^*J*_C-F_ = 12 Hz), 129.6, 135.3, 143.0 (d, ^4^*J*_C-F_ = 2.3 Hz), 155.5 (d, ^1^*J*_C-F_ = 233 Hz), and 168.7 ppm. HRMS: (EI) Calculated for C_25_H_28_BrFN_4_O_2_ (M^+^) = 516.4278. Found: 516.4267.

##### 1-(6-Fluoro-2,3-dihydro-benzo[*b*][1,4]oxazin-4-yl)-2-{4-[3-(5-bromo-1*H*-3-indolyl)-propyl]-1-piperazinyl} ethanamide (**7o**)

To a solution of 3-(5-Bromo-1*H*-3-indolyl)-propyl-4-methylbencensulfonate **1c** (294 mg; 0.72 mmol) in CH_3_CN (50 mL), 1-(6-Fluoro-2,3-dihydro-benzo[*b*][1,4]oxazin-4-yl)-2-(1-piperazinyl) ethanamide **6c** (200 mg; 0.72 mmol) and anhydrous K_2_CO_3_ (100 mg; 0.72 mmol) were added. The crude mixture was column chromatographed to provide pure **7o** (251 mg; 70% yield) as a yellow light solid. m.p.: 97.2–98.8 °C; ^1^H-NMR (DMSO-*d*_6_): δ 1.74 (q, 2H, H-2′, *J* = 6.7 Hz), 2.21–2.52 (m, 10H, H-3′, H-4′ and H-5′), 2.65 (t, 2H, H-1′, *J* = 7.0 Hz), 3.35 (s, 2H, H-7′), 3.92 (m, 2H, H-3″), 4.24 (m, 2H, H-2″), 6.84–6.93 (m, 2H, H-7″ and H8″), 7.10–7.21 (m, 2H, H-2 and H-6), 7.30 (d, 1H, H-7, *J* = 8.6 Hz), 7.68 (s, 1H, H-4), 7.85 (br. d, 1H, H-5″, *J* = 7.8 Hz), and 11.04 (s, 1H, H-1) ppm. ^13^C-NMR (DMSO-*d*_6_): δ 22.4, 27.6, 52.9 (2X), 53.2 (2X), 57.7, 66.1, 110.3 (d, ^2^*J*_C-F_ = 28.5 Hz), 111.2, 112.0 (d, ^2′^*J*_C-F_ = 22.5 Hz), 113.8, 114.7, 117.9 (d, ^3^*J*_C-F_ = 8.8 Hz), 121.1, 123.6, 124.4, 126, 126.9 (d, ^3′^*J*_C-F_ = 12.1 Hz), 128.5, 129.6, 132.2, 135.3, 138.2, 143.0 (d, ^4^*J*_C-F_ = 2.2 Hz), 155.5 (d, ^1^*J*_C-F_ = 233 Hz), and 168.7 ppm. HRMS: (EI) Calculated for C_25_H_28_BrFN_4_O_2_ (M^+^) = 516.4278. Found: 516.4272.

#### 3.1.4. General procedure for the synthesis of 2,3-Dihydro-benzo[1,4]oxazin-4-yl)-2-{4-[3-(1*H*-3-indolyl)-propyl]-1-piperazinyl} ethanamides **7d**–**f** and **7j**–**l**. Method 2

##### Synthesis of 1-(6-Chloro-2,3-dihydro-benzo[*b*][1,4]oxazin-4-yl)-2-{4-[3-(1*H*-3-indolyl)-propyl]-1- piperazinyl} ethanamide. Compound (**7d**) as a Model

To a stirred solution of 3-(3-Piperazin-1-yl-propyl)-1*H*-indol **9a** (224 mg; 0.92 mmol), in dry CH_3_CN (50 mL), 2-Chloro-1-(6-chloro-2,3-dihydro-benzo[*b*][1,4]oxazin-4-yl) ethanamide **4e** (227 mg; 0.92 mmol), and anhydrous K_2_CO_3_ (127 mg; 0.92 mmol) were added. The mixture was heated at 80 °C for 24 h. After this time, the resulting mixture was poured into water (100 mL) and extracted with EtOAc (4 × 50 mL), dried over anhydrous Na_2_SO_4_, and concentrated under reduced pressure. The organic crude was purified by column chromatography EtOAc/MeOH (6:1) to give pure **7d** (290 mg; 71.6% yield) as a yellow light solid. m.p.: 135.3–136.1 °C; ^1^H-NMR (DMSO-*d*_6_): δ 1.78 (q, 2H, H-2′, *J* = 7.0 Hz), 2.20–2.52 (m, 10H, H-3′, H-4′ and H-5′), 2.68 (t, 2H, H-1′, *J* = 7.3 Hz), 3.32 (s, 2H, H-7′), 3.91 (t, 2H, H-3″, *J* = 3.7 Hz), 4.27 (t, 2H, H-2″, *J* = 3.8 Hz), 6.90 (d, 1H, H-8″, *J* = 8.8 Hz), 6.96 (t, 1H, H-5 or H-6, *J* = 7.3 Hz), 7.02–7.12 (m, 3H, 2-H, H-6 or H-5 and H-7″), 7.34 (d, 1H, H-4, *J* = 8.0 Hz), 7.50 (d, 1H, H-7, *J* = 7.8 Hz), 8.09 (br. s, 1H, H-5″), and 10.75 (br. s, 1H, H-1) ppm. ^13^C-NMR (DMSO-*d*_6_): δ 22.9, 27.6, 39.3, 40.5, 52.9 (2X), 53.2 (2X), 58.0, 66.4, 111.8, 114.9, 118.5, 118.6, 118.7, 121.2, 122.6, 123.5, 123.6, 125.2, 127.6, 127.7, 136.8, 145.5, and 168.6 ppm. HRMS: (EI) Calculated for C_25_H_29_ClN_4_O_2_ (M^+^) = 453.9864. Found: 453.9856.

##### 1-(6-Bromo-2,3-dihydro-benzo[*b*][1,4]oxazin-4-yl)-2-{4-[3-(1*H*-3-indolyl)-propyl]-1-piperazinyl} ethanamide (**7e**)

To a solution of 3-(3-Piperazin-1-yl-propyl)-1*H*-indol **9a** (241 mg; 0.99 mmol) in CH_3_CN (50 mL), 2-Chloro-1-(6-Bromo-2,3-dihydro-benzo[*b*][1,4]oxazin-4-yl) ethanamide **4d** (287 mg; 0.99 mmol) and anhydrous K_2_CO_3_ (137 mg; 0.99 mmol) were added to afford **7e** (298 mg; 62.6% yield) as a yellow light solid. m.p.: 132.7–134.1 °C; ^1^H-NMR (DMSO-*d*_6_): δ 1.77 (q, 2H, H-2′, *J* = 6.7 Hz), 2.27–2.51 (m, 10H, H-3′, H-4′ and H-5′), 2.68 (t, 2H, H-1′, *J* = 7.3 Hz), 3.32 (s, 2H, H-7′), 3.91 (m, 2H, H-3″), 4.27 (t, 2H, H-2″, *J* = 3.8 Hz), 6.86 (d, 1H, H-8″, *J* = 8.8 Hz), 6.96 (td, 1H, H-5 or H-6, *J_o_* = 7.3 Hz, *J_m_* = 1.0 Hz), 7.05 (td, 1H, H-6 or H-5, *J_o_* = 7.3 Hz, *J_m_* = 1.0 Hz), 7.10 (d, 1H, H-2, *J* = 1.9 Hz), 7.19 (dd, 1H, H-7″, *J_o_* = 8.7 Hz, *J_m_* = 2.1 Hz), 7.33 (d, 1H, H-4, *J* = 8.0 Hz), 7.50 (d, 1H, H-7, *J* = 7.8 Hz), 8.20 (br. s, 1H, H-5″), and 10.77 (br.s, 1H, H-1) ppm. ^13^C-NMR (DMSO-*d*_6_): δ 22.9, 27.6, 39.3, 40.5, 52.9 (2X), 53.2 (2X), 58.0, 66.5, 111.1, 111.8, 114.8, 118.5, 118.7, 119.1, 121.2, 122.6, 125.4, 126.3, 127.6, 128.1, 136.7, 146.0, and 168.6 ppm. HRMS: (EI) Calculated for C_25_H_29_BrN_4_O_2_ (M^+^) = 498.4373. Found: 498.4364.

##### 1-(6-Methoxy-2,3-dihydro-benzo[*b*][1,4]oxazin-4-yl)-2-{4-[3-(1*H*-3-indolyl)-propyl]-1-piperazinyl} ethanamide (**7f**)

To a solution of 3-(3-Piperazin-1-yl-propyl)-1*H*-indol **9a** (282 mg; 1.16 mmol) in CH_3_CN (50 mL), 2-Chloro-1-(6-Methoxy-2,3-dihydro-benzo[*b*][1,4]oxazin-4-yl) ethanamide **4f** (280 mg; 1.16 mmol) and anhydrous K_2_CO_3_ (160 mg; 1.16 mmol) were added to provide pure **7f** (400 mg; 79% yield) as a yellow light solid. m.p.: 123.6–125.1 °C; ^1^H-NMR (DMSO-*d*_6_): δ 1.75 (q, 2H, H-2′, *J* = 7.0 Hz), 2.23–2.49 (m, 10H, H-3′, H-4′ and H-5′), 2.65 (t, 2H, H-1′, *J* = 7.4 Hz), 3.31 (s, 2H, H-7′), 3.65 (s, 3H, H-6″), 3.86 (t, 2H, H-3″, *J* = 3.8 Hz), 4.18 (t, 2H, H-2″, *J* = 3.8 Hz), 6.63 (dd, 1H, H-7″, *J_o_* = 8.9 Hz, *J_m_* = 2.3 Hz), 6.79 (d, 1H, H8″, *J* = 8.9 Hz), 6.94 (t, 1H, H-5 or H-6, *J* = 7.0 Hz), 7.03 (t, 1H, H-6 or H-5, *J* = 6.9 Hz), 7.08 (d, 1H, H-2, *J* = 1.9 Hz), 7.31 (d, 1H, H-4, *J* = 8.0 Hz), 7.48 (d, 1H, H-7, *J* = 7.8 Hz), 7.61 (br. s, 1H, H-5″), and 10.74 (br. s, 1H, H-1) ppm. ^13^C-NMR (DMSO-*d*_6_): δ 22.8, 27.6, 39.3, 40.5, 53.0 (2X), 53.2 (2X), 55.8, 58.0, 66.1, 109.3, 111.7, 114.8, 117.3, 118.4, 118.7, 121.1, 122.5, 126.8, 127.4, 127.6, 136.7, 140.7, 152.6, and 168.3 ppm. HRMS: (EI) Calculated for C_26_H_32_N_4_O_3_ (M^+^) = 449.5673. Found: 449.5669.

##### 1-(6-Chloro-2,3-dihydro-benzo[*b*][1,4]oxazin-4-yl)-2-{4-[3-(5-fluoro-1*H*-3-indolyl)-propyl]-1-piperazinyl} ethanamide (**7j**)

To a solution of 5-Fluoro-3-(3-piperazin-1-yl-propyl)-1*H*-indol **9b** (340 mg; 1.3 mmol) in CH_3_CN (50 mL), 2-Chloro-1-(6-chloro-2,3-dihydro-benzo[*b*][1,4]oxazin-4-yl) ethanamide **4e** (320 mg; 1.3 mmol) and anhydrous K_2_CO_3_ (180 mg 1.3 mmol) were added to afford pure compound **7j** (540 mg; 91% yield) as a yellow light solid. m.p.: 120.5–122.8 °C; ^1^H-NMR (DMSO-*d*_6_): δ 1.75 (q, 2H, H-2′, *J* = 7.1 Hz), 2.21–2.50 (m, 10H, H-3′, H-4′ and H-5′), 2.64 (t, 2H, H-1′, *J* = 7.4 Hz), 3.33 (s, 2H, H-7′), 3.91 (t, 2H, H-3″,*J* = 3.7 Hz), 4.27 (t, 2H, H-2″, *J* = 3.7 Hz), 6.85–6.92 (m, 2H, H-6 and H-8″), 7.07 (dd, 1H, H-7″, *J_o_* = 8.7 Hz, *J_m_* = 2.3 Hz), 7.19 (d, 1H, H-2, *J* = 1.7 Hz), 7.24 (dd, 1H, H-7, J*_o_*= 10.2 Hz, *J_m_* = 2.3 Hz), 7.31 (dd, 1H, H-4, *J_o_* = 8.9 Hz, *J_m_* = 4.7 Hz), 8.07 (br. s, 1H, H-5″), and 10.88 (br. s, 1H, H-1) ppm. ^13^C-NMR (DMSO-*d*_6_): δ 22.7, 27.5, 39.3, 40.5, 52.9 (2X), 53.2 (2X), 57.8, 66.4, 103.4 (d, ^2^*J*_C-F_ = 23 Hz), 109.3 (d, ^2′^*J*_C-F_ = 26.3 Hz), 112.6 (d, ^3^*J*_C-F_ = 9.9 Hz), 114.4, 115.1 (d, ^4^J_C-F_ = 5.4 Hz), 118.7, 123.5, 124.8, 125.2, 127.5, 127.9 (d, ^3′^*J*_C–F_ = 9.3 Hz), 133.3, 145.5, 157.0 (d, ^1^*J*_C–F_ = 231 Hz), and 168.6 ppm. HRMS: (EI) Calculated for C_25_H_28_ClFN_4_O_2_ (M^+^) = 471.9769. Found: 471.9767.

##### 1-(6-Bromo-2,3-dihydro-benzo[*b*][1,4]oxazin-4-yl)-2-{4-[3-(5-fluoro-1*H*-3-indolyl)-propyl]-1-piperazinyl} ethanamide (**7k**)

To a solution of 5-Fluoro-3-(3-piperazin-1-yl-propyl)-1*H*-indol **9b** (784 mg; 3 mmol) in CH_3_CN (50 mL), 2-Chloro-1-(6-bromo-2,3-dihydro-benzo[*b*][1,4]oxazin-4-yl) ethanamide **4d** (870 mg; 3 mmol) and anhydrous K_2_CO_3_ (180 mg 1.3 mmol) were added to afford pure compound **7k** (1130 mg; 75.3% yield) as a yellow light solid. m.p.: 102.8–103.9 °C; ^1^H-NMR (DMSO-*d*_6_): δ 1.80 (q, 2H, H-2′, *J* = 7.0 Hz), 2.26–2.56 (m, 10H, H-3′, H-4′ and H-5′), 2.69 (t, 2H, H-1′, *J* = 7.3 Hz), 3.38 (s, 2H, H-7′), 3.97 (t, 2H, H-3″,*J* = 3.4 Hz), 4.33 (t, 2H, H-2″, *J* = 3.6 Hz), 6.89–6.99 (m, 2H, H-6, and H-8″), 7.13 (dd, 1H, H-7″, *J_o_* = 8.7 Hz, *J_m_* = 2.3 Hz), 7.24 (d, 1H, H-2, *J* = 1.9 Hz), 7.30 (dd, 1H, H-7, *J_o_* = 10.2 Hz, *J_m_* = 2.3 Hz), 7.37 (dd, 1H, H-4, *J_o_* = 8.8 Hz, *J_m_* = 4.5 Hz), 8.13 (br. s, 1H, H-5″), and 10.93 (br. s, 1H, H-1) ppm. ^13^C-NMR (DMSO-*d*_6_): δ 22.7, 27.5, 39.3, 40.5, 52.9 (2X), 53.2 (2X), 57.9, 66.4, 103.4 (d, ^2^*J*_C-F_ = 23.1 Hz), 109.3 (d, ^2′^*J*_C-F_ = 26 Hz), 112.6 (d, ^3^*J*_C-F_ = 9.9 Hz), 115.1 (d, ^4^*J*_C-F_ = 4.9 Hz), 118.7, 123.4, 123.5, 124.8, 125.3, 127.6, 127.9 (d, ^3′^*J*_C–F_ = 9.3 Hz), 133.3, 145.5, 157.0 (d, ^1^*J*_C–F_ = 231 Hz), and 168.6 ppm. HRMS: (EI) Calculated for C_25_H_28_BrFN_4_O_2_ (M^+^) = 516.4278. Found: 516.4270.

##### 1-(6-Methoxy-2,3-dihydro-benzo[*b*][1,4]oxazin-4-yl)-2-{4-[3-(5-fluoro-1*H*-3-indolyl)-propyl]-1-piperazinyl} ethanamide (**7l**)

To a solution of 5-Fluoro-3-(3-piperazin-1-yl-propyl)-1*H*-indol **9b** (379 mg; 1.45 mmol) in CH_3_CN (50 mL), 2-chloro-1-(6-methoxy-2,3-dihydro-benzo[*b*][1,4] oxazin-4-yl) ethanamide **4f** (350 mg; 1.45 mmol) and anhydrous K_2_CO_3_ (200 mg; 1.45 mmol) were added to afford pure compound **7l** (535 mg; 81.6% yield) as a yellow light solid. m.p.: 94.5–97.3 °C; ^1^H-NMR (DMSO-*d*_6_): δ 1.75 (q, 2H, H-2′, *J* = 7.0 Hz), 2.2–2.50 (m, 10H, H-3′, H-4′ and H-5′), 2.64 (t, 2H, H-1′, *J* = 7.4 Hz), 3.34 (s, 2H, H-7′), 3.68 (s, 3H, H-6″), 3.89 (t, 2H, H-3″,*J* = 4.5 Hz), 4.21 (t, 2H, H-2″, *J* = 3.9 Hz), 6.65 (dd, 1H, H-7″, *J_o_* = 8.9 Hz, *J_m_* = 2.9 Hz), 6.81 (d, 1H, H-8″, *J* = 8.9 Hz), 6.89 (td, 1H, H-6, *J_o_* = 9.2 Hz, *J_m_* = 2.5 Hz), 7.19 (d,1H, H-2, *J* = 2.2 Hz), 7.25 (dd, 1H, H-7, *J_o_* = 10.1 Hz, *J_m_* = 2.5 Hz), 7.32 (dd, 1H, H-4, *J_o_* = 8.8 Hz, *J_m_* = 4.6 Hz), 7.62 (br. s, 1H, H-5″), and 10.88 (br. s, 1H, H-1) ppm. ^13^C-NMR (DMSO-*d*_6_): δ 22.7, 27.6, 39.3, 40.5, 53.1 (2X), 53.3 (2X), 55.9, 57.9, 66.2, 103.4 (d, ^2^*J*_C-F_ = 23.2 Hz), 109.3 (d, ^2′^*J*_C-F_ = 26 Hz), 109.3, 112.6 (d, ^3^*J*_C-F_ = 10 Hz), 115.2 (d, ^4^*J*_C-F_ = 4.4 Hz), 117.4, 124.9, 126.9, 127.9 (d, ^3′^*J*_C–F_ = 10 Hz), 128.6, 133.4, 140.7, 152.7, 157.0 (d, ^1^*J*_C–F_ = 231 Hz), and 168.4 ppm. HRMS: (EI) Calculated for C_26_H_31_FN_4_O_3_ (M^+^) = 467.5577. Found: 467.5567.

#### 3.1.5. General Procedure for the Synthesis of *N*-(2-morpholin-4-yl-ethyl)-benzamides nitro-fluorinated Derivatives **10a**–**d**

##### 4-Fluoro-*N*-(2-morpholin-4-yl-ethyl)-2-nitro-benzamide (**10a**) as a Model

To a solution of 4-Fluoro-2-nitro-benzoyl chloride (828 mg; 4.07 mmol) in dry THF (50 mL), 2-morpholin-4-yl-ethylamine (0.53 mL; 4.07 mmol) was added. The mixture was stirred at 0 °C under nitrogen atmosphere for 2 h. After this time, saturated solution of NaHCO_3_ was added (100 mL) and the mixture was extracted with EtOAc (3 × 60 mL). The organic layers were dried over anhydrous Na_2_SO_4_ filtered, and concentrated under vacuum conditions to give a crude form, which was purified by column chromatography with EtOAc/MeOH (6:1) as eluent to give **10a** (963 mg; 80% yield) as a yellow light solid. m.p.: 149.8–151.5 °C; ^1^H-NMR (acetone-*d*_6_): δ 2.32 (t, 4H, H-4′, *J* = 4.6 Hz), 2.42 (t, 2H, H-3′, *J* = 6.3 Hz), 3.35 (q, 2H, H-2′, *J* = 6.3 Hz), 3.46 (t, 4H, H-5′, *J* = 4.5 Hz), 7.45 (td, 1H, H-5, *J_o_* = 8.2 Hz, *J_m_* = 2.5 Hz), 7.59 (dd, 1H, H-6, *J_o_* = 8.5 Hz, *J_m_* = 5.5 Hz), 7.65 (br. s, 1H, H-1′), and 7.70 (dd, 1H, H-3, *J_o_* = 8.5 Hz, *J_m_* = 2.6 Hz) ppm. ^13^C-NMR (acetone-*d*_6_): δ 36.1, 53 (2X), 56.6, 66 (2X), 111.3 (d, 2*J*_C-F_ = 27 Hz), 119.6 (d, 2′*J*_C-F_ = 22 Hz), 129.1 (d, 4*J*_C-F_ = 3.3 Hz), 130.5 (d, 3*J*_C-F_ = 9 Hz), 148.1 (d, 3′*J*_C-F_ = 8.2 Hz), 161.7 (d, 1*J*_C-F_ = 251 Hz), and 164.2 ppm. HRMS: (EI) Calculated for C_13_H_16_FN_3_O_4_ (M^+^) = 298.1203. Found: 298.1198.

##### 4-Fluoro-*N*-(2-morpholin-4-yl-ethyl)-3-nitro-benzamide (**10b**)

4-Fluoro-3-nitro-benzoyl chloride (553 mg; 2.72 mmol), and 2-morpholin-4-yl-ethylamine (0.35 mL; 2.72 mmol), to afford **10b** (712 mg; 88% yield) as a yellow light solid. m.p.: 132.5–133.5 °C; ^1^H-NMR (acetone-*d*_6_): δ 2.32 (t, 4H, H-4′, *J* = 4.4 Hz), 2.43 (t, 2H, H-3′, *J* = 6.6 Hz), 3.37–3.50 (m, 6H, H-2′ and H-5′), 7.48 (m, 1H, H-5), 7.92 (br. s, 1H, H-1′), 8.16 (m, 1H, H-2), and 8.45 (dd, 1H, H-6, *J_o_* = 7.2 Hz, *J_m_* = 2.3 Hz) ppm. ^13^C-NMR (acetone-*d*_6_): δ 36.4, 53.1 (2X), 56.9, 66.1 (2X), 118.1 (d, ^2^*J*_C-F_ = 21.4 Hz), 124.6 (d, ^4^*J*_C-F_ = 1.6 Hz), 131.6 (d, ^3^*J*_C-F_ = 3.8 Hz), 134.2 (d, ^2′^*J*_C-F_ = 9.9 Hz), 150.4 (d, ^3′^*J*_C-F_ = 3.9 Hz), 156.2 (d, ^1^J_C-F_ = 266 Hz), and 163 ppm. HRMS: (EI) Calculated for C_13_H_16_FN_3_O_4_ (M^+^) = 298.1203, Found: 298.1201.

##### 5-Fluoro-*N*-(2-morpholin-4-yl-ethyl)-2-nitro-benzamide (**10c**)

5-Fluoro-2-nitro-benzoyl chloride (803 mg; 3.94 mmol), and 2-morpholin-4-yl-ethylamine (0.51 mL; 3.94 mmol), to afford **10c** (951 mg; 81% yield) as a yellow light solid. m.p.: 103.4–104.5 °C; ^1^H-NMR (acetone-*d*_6_): δ 2.33 (t, 4H, H-4′, *J* = 4.6 Hz), 2.44 (t, 2H, H-3′, *J* = 6.3 Hz), 3.36 (m, 2H, H-2′), 3.46 (t, 4H, H-5′, *J* = 4.5 Hz), 7.23–7.37 (m, 2H, H-3, and H-4), 7.67 (br. s, 1H, H-1′), and 8.02 (dd, 1H, H-6, *J_o_* = 9.1 Hz, *J_m_* = 4.8 Hz) ppm. ^13^C-NMR (acetone-*d*_6_): δ 36.1, 53 (2X), 56.5, 66 (2X), 115.6 (d, ^2^*J*_C-F_ = 25.3 Hz), 116.5 (d, ^2′^*J*_C-F_ = 23.6 Hz), 126.8 (d, ^3^*J*_C-F_ = 9.9 Hz), 136.0 (d, ^3′^*J*_C-F_ = 8.8 Hz), 143 (d, ^4^*J*_C-F_ = 3.3 Hz), 163.9, and 163.9 (d, ^1^*J*_C-F_ = 256 Hz) ppm. HRMS: (EI) Calculated for C_13_H_16_FN_3_O_4_ (M^+^) = 298.1203. Found: 298.1201.

##### 2-Fluor-*N*-(2-morpholin-4-yl-ethyl)-5-nitro-benzamide (**10d**)

2-Fluoro-5-nitro-benzoyl chloride (825 mg; 4.05 mmol), and 2-morpholin-4-yl-ethylamine (0.53 mL; 4.05 mmol), to afford **10d** (902 mg; 75% yield) as a yellow light solid. m.p.: 126.4–127.2 °C; ^1^H-NMR (acetone-*d*_6_): δ 2.35 (t, 4H, H-4′, *J* = 4.5 Hz), 2.46 (t, 2H, H-3′, *J* = 6.3 Hz), 3.43 (q, 2H, H-2′, *J* = 6.5 Hz), 3.49 (t, 4H, H-5′, *J* = 4.5 Hz), 7.43 (m, 1H, H-3), 7.66 (br. s, 1H, H-1′), 8.30 (m, 1H, H-4), and 8.56 (dd, 1H, H-6, *J_o_* = 6.3 Hz, *J_m_* = 3 Hz) ppm. ^13^C-NMR (acetone-*d*_6_): δ 36.1, 52.9 (2X), 56.2, 66.1 (2X), 117.4 (d, ^2^*J*_C-F_ = 26.9 Hz), 123.6 (d, ^2′^*J*_C-F_ = 16.5 Hz), 126.3 (d, ^4^*J*_C-F_ = 4.9 Hz), 127.5 (d, ^3^*J*_C-F_ = 11 Hz), 144(d, ^3′^*J*_C-F_ = 9 Hz), 160.4, and 162.8 (d, ^1^*J*_C-F_ = 259 Hz) ppm. HRMS: (EI) Calculated for C_13_H_16_FN_3_O_4_ (M^+^) = 298.1203. Found: 298.1201.

#### 3.1.6. General Procedure for the Synthesis of *N*-(2-morpholin-4-yl-ethyl)-benzamides amino-fluorinated Derivatives **11a**–**d**

##### 2-Amino-*N*-(2-morpholin-4-yl-ethyl)-4-fluoro-benzamide (**11a**) as a Model

To a mixture containing water-acetic acid-ethanol (1:1:1), 4-Fluoro-*N*-(2-morpholin-4-yl-ethyl)-2- nitro-benzamide **10a** (1 g; 3.36 mmol) and iron powder (734 mg; 13.1 mmol) were added. The resulting mixture was heated and stirred for 3 h at 70 °C. After this time, the mixture was filtered to remove excess metallic iron, transferred to a flask containing a mixture of EtOAc/H_2_O (400 mL, 1:1), and neutralized with NaHCO_3_ (10 gr). The aqueous phase was extracted with EtOAc (50 mL × 3). The organic layer was dried over anhydrous Na_2_SO_4_ and concentrated under vacuum to give a crude, which was purified by column chromatography with EtOAc/MeOH (6:1) to give **11a** (891 mg; 98% yield) as a yellow light solid. m.p.: 120.3–121.7 °C; ^1^H-NMR (CDCl_3_): δ 2.46 (t, 4H, H-4′, *J* = 4.4 Hz), 2.55 (t, 2H, H-3′, *J* = 6.1 Hz), 3.46 (q, 2H, H-2′, *J* = 5.6 Hz), 3.69 (t, 4H, H-5′, *J* = 4.7 Hz), 5.72 (br. s, 2H, H-2), 6.28–6.35 (m, 2H, H-3 and H-5), 6.57 (br. s, 1H, H-1′), and 7.25 (dd, 1H, H-6, *J_o_* = 9.3 Hz, *J_m_* = 3.2 Hz) ppm. ^13^C-NMR (CDCl_3_): δ 36.7, 53.3 (2X), 56.7, 66.9 (2X), 102.3 (d, ^2^*J*_C-F_ = 24.2 Hz), 103.9 (d, ^2′^*J*_C-F_ = 23 Hz), 112.4 (d, ^4^*J*_C-F_ = 2.2 Hz), 129.3 (d, ^3^*J*_C-F_ = 11 Hz), 151.1 (d, ^3′^*J*_C-F_ = 11.7 Hz), 165.3 (d, ^1^*J*_C-F_ = 249 Hz), and 168.6 ppm. HRMS: (EI) Calculated for C_13_H_18_FN_3_O_2_ (M^+^) = 268.1461. Found: 268.1454.

##### 3-Amino-*N*-(2-morpholin-4-yl-ethyl)-4-fluoro-benzamide (**11b**)

4-Fluoro-*N*-(2-morpholin-4-yl-ethyl)-3-nitro-benzamide **10b** (1 g; 3.36 mmol) and iron powder (734 mg; 13.1 mmol) to afford **11b** (726 mg; 81% yield) as a yellow light solid. m.p.: 147.4–149.1 °C; ^1^H-NMR (DMSO-*d*_6_): δ 2.40–2.51 (m, 4H, H-4′), 2.57 (br. s, 2H, H-3′), 3.40 (q, 2H, H-2′, *J* = 6.6 Hz), 3.63 (t, 4H, H-5′, *J* = 4.3 Hz), 5.39 (br. s, 2H, H-3), 6.99–7.15 (m, 2H, H-2 and H-4), 7.31 (dd, 1H, H-6, *J_o_* = 8.9 Hz, *J_m_* = 1.6 Hz), and 6.57 (br. t, 1H, H-1′, *J* = 5.2 Hz), ppm. ^13^C-NMR (DMSO-*d*_6_): δ 36.9, 53.7 (2X), 57.8, 66.6 (2X), 115.0 (d, ^2^*J*_C-F_ = 30 Hz), 115.1 (d, ^3^*J*_C-F_ = 3.3 Hz), 116 (d, ^3′^*J*_C-F_ = 6 Hz), 131.7 (d, ^4^*J*_C-F_ = 2.8 Hz), 136.7 (d, ^2′^*J*_C-F_ = 13.2 Hz), 152.5 (d, ^1^*J*_C-F_ = 242 Hz), and 168.3 ppm. HRMS: (EI) Calculated for C_13_H_18_FN_3_O_2_ (M^+^) = 268.1461. Found: 268.1458.

##### 2-Amino-*N*-(2-morpholin-4-yl-ethyl)-5-fluoro-benzamide (**11c**)

5-Fluoro-*N*-(2-morpholin-4-yl-ethyl)-2-nitro-benzamide **10c** (1 g; 3.36 mmol) and iron powder (734 mg; 13.1 mmol) to afford **11c** (697 mg; 78% yield) as a yellow light solid. m.p.: 104.8–105.6 °C; ^1^H-NMR (CDCl_3_): δ 2.47 (t, 4H, H-4′, *J* = 4.4 Hz), 2.55 (t, 2H, H-3′, *J* = 6.1 Hz), 3.47 (q, 2H, H-2′, *J* = 5.6 Hz), 3.69 (t, 4H, H-5′, *J* = 4.6 Hz), 5.28 (br. s, 2H, H-2), 6.55–6.63 (br. m, 2H, H-3, and H-1′), 6.93 (td, 1H, H-6, *J_o_* = 8.6 Hz, *J_m_* = 2.9 Hz), and 7.0 (dd, 1H, H-4, *J_o_* = 9.1 Hz, *J_m_* = 2.7 Hz) ppm. ^13^C-NMR (CDCl_3_): δ 35.9, 53.3 (2X), 56.8, 67 (2X), 113.1 (d, ^2^*J*_C-F_ = 23 Hz), 116.6 (d, ^3^*J*_C-F_ = 5.1 Hz), 118.4 (d, ^3′^*J*_C-F_ = 7.3 Hz), 119.5 (d, ^2′^*J*_C-F_ = 23 Hz), 144.8 (d, ^4^*J*_C-F_ = 1.5 Hz), and 155.5 (d, ^1^*J*_C-F_ = 236 Hz), 168.3 ppm. HRMS: (EI) Calculated for C_13_H_18_FN_3_O_2_ (M^+^) = 268.1461. Found: 268.1459.

##### 5-Amino-*N*-(2-morpholin-4-yl-ethyl)-2-fluoro-benzamide (**11d**)

2-Fluoro-*N*-(2-morpholin-4-yl-ethyl)-5-nitro-benzamide **10d** (1 g; 3.36 mmol) and iron powder (734 mg; 13.1 mmol) to afford **11d** (798 mg; 89% yield) as a yellow light solid. m.p.: 106.8–107.9 °C; ^1^H-NMR (CDCl_3_): δ 2.37 (t, 4H, H-4′, *J* = 4.4 Hz), 2.45 (t, 2H, H-3′, *J* = 6.1 Hz), 3.24–3.50 (br. m, 4H, H-5 H-2′), 3.59 (t, 4H, H-5′, *J* = 4.7 Hz), 6.59 (m, 1H, H-6), 6.78 (m, 1H, H-3), 7.23 (dd, 1H, H-4, *J_o_* = 6.4 Hz, *J_m_* = 3.2 Hz), and 7.28 (br. s, 1H, H-1′) ppm. ^13^C-NMR (CDCl_3_): δ 36.3, 53.2 (2X), 56.5, 67 (2X), 116.6 (d, ^2^*J*_C-F_ = 26.4 Hz), 117.0 (d, ^4^*J*_C-F_ = 1.5 Hz), 119.1 (d, ^3^*J*_C-F_ = 8.8 Hz), 121.3 (d, ^2′^*J*_C-F_ = 13.2 Hz), 143.1 (d, ^3′^*J*_C-F_ = 2.2 Hz), 154.2 (d, ^1^*J*_C-F_ = 238 Hz), and 163.4 ppm. HRMS: (EI) Calculated for C_13_H_18_FN_3_O_2_ (M^+^) = 268.1461. Found: 268.1457.

#### 3.1.7. General Procedure for the Synthesis of (2-Chloro-acetylamino)-*N*-(2-morpholin-4-yl-ethyl) fluorinated Benzamides Derivatives **12a**–**d**

##### 2-(2-Chloro-acetylamino)-4-fluoro-*N*-(2-morpholin-4-yl-ethyl)-benzamide (**12a**) as a Model

To a solution of 2-amino-*N*-(2-morpholin-4-yl-ethyl)-4-fluoro-benzamide **11a** (400 mg; 1.5 mmol) in dry THF (60 mL), 2-chloro-acetylchloride (0.12 mL; 1.5 mmol) was added. The mixture was stirred at 0 °C under nitrogen atmosphere for 2 h. After this time, a saturated solution of NaHCO_3_ (100 mL) was added. The mixture was extracted with EtOAc (3 X 100 mL). The organic layers were dried over anhydrous Na_2_SO_4_, filtered, and concentrated under vacuum conditions to give a crude, which was purified by column chromatography EtOAc/MeOH (6:1), to give **12a** (479 mg; 93% yield) as a white solid. m.p.: 124.8–126.3 °C; ^1^H-NMR (CDCl_3_): δ 2.48 (t, 4H, H-4′, *J* = 4.4 Hz), 2.58 (t, 2H, H-3′, *J* = 5.9 Hz), 3.51 (q, 2H, H-2′, *J* = 5.4 Hz), 3.69 (t, 4H, H-5′, *J* = 4.6 Hz), 4.13 (s, 2H, H-1″), 6.81 (td, 1H, H-5, *J_o_* = 7.7 Hz, *J_m_* = 2.7 Hz), 6.88 (br. s, 1H, H-1′), 7.46 (dd, 1H, H-5, *J_o_* = 8.8 Hz, *J_m_* = 6.1 Hz), 8.42 (dd, 1H, H-3, *J_o_* = 11.6 Hz, *J_m_* = 2.7 Hz), and 12.24 (br. s, 1H, H-2) ppm. ^13^C-NMR (CDCl_3_): δ 36, 43.2, 53.3 (2X), 56.5, 66.9 (2X), 108.7 (d, ^2^*J*_C-F_ = 28 Hz), 110.6 (d, ^2′^*J*_C-F_ = 22 Hz), 116.8 (d, ^4^*J*_C-F_ = 3.7 Hz), 128.4 (d, ^3^*J*_C-F_ = 10.3 Hz), 141 (d, ^3′^*J*_C-F_ = 12.5 Hz), 164.7 (d, ^1^*J*_C-F_ = 252 Hz), 165.5, and 167.8 ppm. HRMS: (EI) Calculated for C_15_H_19_ClFN_3_O_3_ (M^+^) = 344.1177. Found: 344.1173.

##### 3-(2-Chloro-acetylamino)-4-fluoro-*N*-(2-morpholin-4-yl-ethyl)-benzamide (**12b**)

3-Amino-*N*-(2-morpholin-4-yl-ethyl)-4-fluoro-benzamide **11b** (400 mg; 1.5 mmol) and 2-chloro-acetylchloride (0.12 mL; 1.5 mmol), to afford **12b** (482 mg; 94% yield) as a white solid. m.p.: 129.1–130.8 °C; ^1^H-NMR (CDCl_3_): δ 2.48 (t, 4H, H-4′, *J* = 4.4 Hz), 2.57 (t, 2H, H-3′, *J* = 6.1 Hz), 3.50 (q, 2H, H-2′, *J* = 5.6 Hz), 3.71 (t, 4H, H-5′, *J* = 4.7 Hz), 4.20 (s, 2H, H-1″), 6.89 (br. s, 1H, H-1′), 7.14–7.19 (m, 1H, H-5), 7.64–7.68 (m, 1H, H-2), 8.57 (br. s, 1H, H-3), and 8.67 (dd, 1H, H-6, *J_o_* = 7.3 Hz, *J_m_* = 2 Hz) ppm. ^13^C-NMR (CDCl_3_): δ 36.2, 42.9, 53.3 (2X), 56.6, 66.9 (2X), 115.4 (d, ^2^*J*_C-F_ = 19.8 Hz), 119.5 (d, ^4^*J*_C-F_ = 1.5 Hz), 125.2 (d, ^3^*J*_C-F_ = 8.8 Hz), 125.4 (d, ^2′^*J*_C-F_ = 11 Hz), 131.4 (d, ^3′^*J*_C-F_ = 2.9 Hz), 154.3 (d, ^1^*J*_C-F_ = 250 Hz), 164.1, and 166 ppm. HRMS: (EI) Calculated for C_15_H_19_ClFN_3_O_3_ (M^+^) = 344.1177. Found: 344.1176.

##### 2-(2-Chloro-acetylamino)-5-fluoro-*N*-(2-morpholin-4-yl-ethyl)-benzamide (**12c**)

2-Amino-*N*-(2-morpholin-4-yl-ethyl)-5-fluoro-benzamide **11c** (400 mg; 1.5 mmol) and 2-chloro-acetylchloride (0.12 mL; 1.5 mmol), to afford **12c** (496 mg, 97% yield) as a white solid. m.p.: 98.5–99.8 °C; ^1^H-NMR (CDCl_3_): δ 2.55 (t, 4H, H-4′, *J* = 4.4 Hz), 2.65 (t, 2H, H-3′, *J* = 6.1 Hz), 3.57 (c, 2H, H-2′, *J* = 5.6 Hz), 3.77 (t, 4H, H-5′, *J* = 4.7 Hz), 4.20 (s, 2H, H-1″), 6.93 (br. s, 1H, H-1′), 7.19–7.27 (m, 2H, H-4 and H-6), 8.61 (dd, 1H, H-3, *J_o_* = 8.9 Hz, *J_m_* = 5.1 Hz), and 11.8 (br. s, 1H, H-2) ppm. ^13^C-NMR (CDCl_3_): δ 36.1, 43.1, 53.3 (2X), 56.5, 66.9 (2X), 113.3 (d, ^2^*J*_C-F_ = 24 Hz), 119.2 (d, ^4^*J*_C-F_ = 22 Hz), 122.7 (d, ^3^*J*_C-F_ = 5.9 Hz), 123.6 (d, ^2′^*J*_C-F_ = 7.3 Hz), 134.8 (d, ^3′^*J*_C-F_ = 2.9 Hz), 158.2 (d, ^1^*J*_C-F_ = 245 Hz), 165.1, and 167.3 ppm. HRMS: (EI) Calculated for C_15_H_19_ClFN_3_O_3_ (M^+^) = 344.1177. Found: 344.1164.

##### 5-(2-Chloro-acetylamino)-2-fluoro-*N*-(2-morpholin-4-yl-ethyl)-benzamide (**12d**)

5-Amino-*N*-(2-morpholin-4-yl-ethyl)-2-fluoro-benzamide **11d** (400 mg; 1.5 mmol) and 2-chloro-acetylchloride (0.12 mL; 1.5 mmol), to afford **12d** (483 mg; 94% yield) as a white solid. m.p.: 144.1–145.1 °C; ^1^H-NMR (CDCl_3_): δ 2.48 (t, 4H, H-4′, *J* = 4.2 Hz), 2.57 (t, 2H, H-3′, *J* = 5.9 Hz), 3.54 (q, 2H, H-2′, *J* = 5.4 Hz), 3.69 (t, 4H, H-5′, *J* = 4.4 Hz), 4.15 (s, 2H, H-1″), 7.05–7.13 (m, 1H, H-3), 7.47 (br. s, 1H, H-1′), 7.9 (dd, 1H, H-4, *J_o_* = 6.5 Hz, *J_m_* = 2.7 Hz), 8.03–8.09 (m, 1H, H-6), and 8.70 (br. s, 1H, H-5) ppm. ^13^C-NMR (CDCl_3_): δ 36.4, 42.9, 53.2 (2X), 56.3, 67 (2X), 116.9 (d, ^2^*J*_C-F_ = 26.4 Hz), 121.5 (d, ^2′^*J*_C-F_ = 13.2 Hz), 123.1 (d, ^4^*J*_C-F_ = 2.2 Hz), 125.2 (d, ^3^*J*_C-F_ = 8.8 Hz), 133.9 (d, ^3′^*J*_C-F_ = 2.9 Hz), 157.4 (d, ^1^*J*_C-F_ = 246 Hz), 162.6, and 164.4 ppm. HRMS: (EI) Calculated for C_15_H_19_ClFN_3_O_3_ (M^+^) = 344.1177. Found: 344.1173.

#### 3.1.8. General Procedure for the Synthesis of (2-{4-[3-(1*H*-3-indolyl)-propyl]-1-piperazinyl}-acetylamino)-*N*-(2-morpholin-4-yl-ethyl)-fluorinated Benzamides Derivatives **13a**–**l**

##### 4-Fluoro-2-(2-{4-[3-(5-fluor-1*H*-3-indolyl)-propyl]-1-piperazinyl}-acetylamino)-*N*-(2-morpholin-4-yl-ethyl) Benzamide (**13a**) as a Model

To a stirred solution of 5-fluoro-3-(3-piperazin-1-yl-propyl)-1*H*-indole **9b** (152 mg; 0.58 mmol), in dry CH_3_CN (50 mL), 2-(2-chloro-acetylamino)-4-fluoro-*N*-(2-morpholin-4-yl-ethyl)-benzamide **12a** (199 mg; 0.58 mmol) and anhydrous K_2_CO_3_ (80 mg; 0.58 mmol) were added. The mixture was heated at 80 °C for 24 h. After this time, the resulting mixture was poured into water (100 mL), extracted with EtOAc (4 × 50 mL), dried over anhydrous Na_2_SO_4_, and concentrated under reduced pressure. The organic crude was purified by column chromatography EtOAc/MeOH (6:1) to give **13a** (298 mg; 62.6%) as a yellow light solid. m.p.: 91.3–93.1 °C; ^1^H-NMR (DMSO-*d*_6_): δ 1.78 (q, 2H, H-2′, *J* = 7.4 Hz), 2.26–2.56 (m, 16H, H-3′, H-4′, H-5′, H-9″ and H-1‴), 2.66 (t, 2H, H-1′, *J* = 7.3 Hz), 3.13 (s, 2H, H-6′), 3.45–3.62 (m, 6H, H-8′, and H-2‴), 6.90 (td, 1H, H-6, *J_o_* = 9.2 Hz, *J_m_* = 2.5 Hz), 7.01 (td, 1H, H-5″, *J_o_* = 8.4 Hz, *J_m_* = 2.7 Hz), 7.20 (d, 1H, H-2, *J* = 2.1 Hz), 7.25 (dd, 1H, H-7, *J_o_* = 10.2 Hz, *J_m_* = 2.5 Hz), 7.32 (dd, 1H, H-4, *J_o_* = 8.8 Hz, *J_m_* = 4.7 Hz), 7.68–7.76 (m, 1H, H-3″), 8.40 (dd, 1H, H-6″, *J_o_* = 12.3 Hz, *J_m_* = 2.6 Hz), 8.62 (t, 1H, H-7″, *J* = 5.5 Hz), 10.87 (s, 1H, H-1), and 12.24 (br. s, 1H, H-7′) ppm. ^13^C-NMR (DMSO-*d*_6_): δ 22.2, 27.0, 36.4, 52.4 (2X), 53.0 (2X), 53.2 (2X), 57.1, 57.5, 61.8, 66.1 (2X), 102.7 (d, ^2^*J*_C-F_ = 23.1 Hz), 106.5 (d, ^2′^*J*_C-F_ = 28.2 Hz), 108.8 (d, ^2″^*J*_C-F_ = 26 Hz), 109.1 (d, ^2‴^*J*_C-F_ = 22.3 Hz), 112.1 (d, ^3^*J*_C-F_ = 9.5 Hz) 114.7 (d, ^4^*J*_C-F_ = 4.8 Hz), 118.2 (d, ^4′^*J*_C-F_ = 2.9 Hz), 124.3, 127.4 (d, ^3′^*J*_C-F_ = 9.5 Hz), 130.2 (d, ^3″^*J*_C–F_ = 9.9 Hz), 132.9, 140.2 (d, ^3‴^*J*_C-F_ = 12.1 Hz), 156.5 (d, ^1^*J*_C–F_ = 231 Hz), 163.3 (d, ^1′^*J*_C-F_ = 246 Hz), 166.8, and 169.8 ppm. HRMS: (EI) Calculated for C_30_H_38_F_2_N_6_O_3_ (M^+^) = 569.3051. Found: 569.3050.

##### 4-Fluoro-3-(2-{4-[3-(5-fluor-1*H*-3-indolyl)-propyl]-1-piperazinyl}-acetylamino)-*N*-(2-morpholin-4-yl-ethyl) Benzamide (**13b**)

5-Fluoro-3-(3-piperazin-1-yl-propyl)-1*H*-indole **9b** (152 mg; 0.58 mmol), 3-(2-chloro-acetylamino)- 4-fluor-*N*-(2-morpholin-4-yl-ethyl)-benzamide **12b** (199 mg; 0.58 mmol), and anhydrous K_2_CO_3_ (80 mg; 0.58 mmol), to afford **13b** (150 mg; 47% yield) as a yellow light solid. m.p.: 92.2–93.9 °C; ^1^H-NMR (DMSO-*d*_6_): δ 1.78 (q, 2H, H-2′, *J* = 7.4 Hz), 2.27–2.56 (m, 16H, H-3′, H-4′, H-5′, H-9″ and H-1‴), 2.66 (t, 2H, H-1′, *J* = 7.3 Hz), 3.19 (s, 2H, H-6′), 3.33–3.36 (m, 2H, H-8′), 3.57 (t, 4H, H-2‴, *J* = 4.4 Hz), 6.89 (td, 1H, H-6, *J_o_* = 9.2 Hz, *J_m_* = 2.5 Hz), 7.20 (d, 1H, H-2, *J* = 1.9 Hz), 7.26 (dd, 1H, H-7, *J_o_* = 10.1 Hz, *J_m_* = 2.4 Hz), 7.29–7.42 (m, 2H, H-4 and H-5″), 7.59–7.67 (m, 1H, H-2″), 8.40–8.50 (m, 2H, H-6″, and H-7″), 9.68 (br. s, 1H, H-7′), and 10.87 (s, 1H, H-1) ppm. ^13^C-NMR (DMSO-*d*_6_): δ 22.7, 27.5, 37.1, 53.3 (2X), 53.4 (2X), 53.7 (2X), 57.7, 57.9, 61.6, 66.7 (2X), 103.4 (d, ^2^*J*_C-F_ = 23.5 Hz), 109.3 (d, ^2″^*J*_C-F_ = 26.4 Hz), 112.6 (d, ^3′^*J*_C-F_ = 9.5 Hz), 115.2 (d, ^4^*J*_C-F_ = 4.4 Hz), 115.6 (d, ^2′^*J*_C-F_ = 19.8 Hz), 123 (d, ^4′^*J*_C-F_ = 6.6 Hz), 124.5 (d, ^3^*J*_C-F_ = 8.1 Hz), 124.9, 126.2 (d, ^2‴^*J*_C-F_ = 11.7 Hz), 127.9 (d, ^3′^*J*_C–F_ = 9.5 Hz), 131.6 (d, ^3^*J*_C-F_ = 2.9 Hz), 133.4, 155.3 (d, ^1^*J*_C-F_ = 248 Hz), 157.0 (d, ^1′^*J*_C–F_ = 230.4 Hz). 165.6, and 169.1 ppm. HRMS: (EI) Calculated for C_30_H_38_F_2_N_6_O_3_ (M^+^) = 569.3051. Found: 569.3046.

##### 2-Fluoro-5-(2-{4-[3-(5-fluor-1*H*-3-indolyl)-propyl]-1-piperazinyl}-acetylamino)-*N*-(2-morpholin-4-yl-ethyl) Benzamide (**13c**)

5-Fluoro-3-(3-piperazin-1-yl-propyl)-1*H*-indole **9b** (152 mg; 0.58 mmol), 5-(2-Chloro-acetylamino)- 2-fluoro-*N*-(2-morpholin-4-yl-ethyl)-benzamide **12d** (199 mg; 0.58 mmol), and anhydrous K_2_CO_3_ (80 mg; 0.58 mmol), to afford **13c** (178 mg; 55% yield) as a yellow light solid. m.p.: 91.8–93.2 °C; ^1^H-NMR (DMSO-*d*_6_): δ 1.76 (q, 2H, H-2′, *J* = 7.4 Hz), 2.32 (t, 2H, H-3′, *J* = 6.9 Hz), 2.36–2.56 (m, 14H, H-4′, H-5′, H-9″ and H-1‴), 2.65 (t, 2H, H-1′, *J* = 7.3 Hz), 3.11 (s, 2H, H-6′), 3.36–3.42 (m, 2H, H-8″), 3.57 (t, 4H, H-2‴, *J* = 4.5 Hz), 6.89 (td, 1H, H-6, *J_o_* = 8.2 Hz, *J_m_* = 2.6 Hz), 7.17–7.28 (m, 3H, H-2, H-7 and H-3″), 7.31 (dd, 1H, H-4, *J_o_* = 8.8 Hz, *J_m_* = 4.6 Hz), 7.73–7.81 (m, 1H, H-6″), 7.92 (dd, 1H, H-4″, *J_o_* = 6.5 Hz, *J_m_* = 2.8 Hz), 8.13–8.24 (m, 1H, H-7″), 9.90 (br. s, 1H, H-7′), and 10.89 (s, 1H, H-1) ppm. ^13^C-NMR (DMSO-*d*_6_): δ 22.7, 27.6, 37, 53.1 (2X), 53.4 (2X), 53.7 (2X), 57.4, 57.9, 62.3, 66.7 (2X), 103.4 (d, ^2^*J*_C-F_ = 22.7 Hz), 109.3 (d, ^2′^*J*_C-F_ = 26.5 Hz), 112.6 (d, ^3″^*J*_C-F_ = 9.9 Hz), 115.2 (d, ^4^*J*_C-F_ = 4.4 Hz), 116.7 (d, ^2″^*J*_C-F_ = 23.8 Hz), 121.33 (d, ^4′^*J*_C-F_ = 2.2 Hz), 123.6 (d, ^2‴^*J*_C-F_ = 8.3 Hz), 124.2 (d, ^3^*J*_C-F_ = 9.5 Hz), 124.9, 127.9 (d, ^3‴^*J*_C–F_ = 9.4 Hz), 133.6, 135.5 (d, ^3′^*J*_C-F_ = 3.3 Hz), 155.4 (d, ^1^*J*_C–F_ = 245 Hz), 157 (d, ^1′^*J*_C-F_ = 231 Hz), 163.7, and 168.9 ppm. HRMS: (EI) Calculated for C_30_H_38_F_2_N_6_O_3_ (M^+^) = 569.3051. Found: 569.3047.

##### 5-Fluoro-2-(2-{4-[3-(5-fluoro-1*H*-3-indolyl)-propyl]-1-piperazinyl}-acetylamino)-*N*-(2-morpholin-4-yl-ethyl) Benzamide (**13d**)

5-Fluoro-3-(3-piperazin-1-yl-propyl)-1*H*-indole **9b** (152 mg; 0.58 mmol), 2-(2-chloro- acetylamino)-5-fluoro-*N*-(2-morpholin-4-yl-ethyl)-benzamide **12c** (199 mg; 0.58 mmol), and anhydrous K_2_CO_3_ (80 mg; 0.58 mmol), to afford **13d** (188 mg; 59% yield) as a yellow light solid. m.p.: 94.2–95.4 °C; ^1^H-NMR (DMSO-*d*_6_): δ 1.79 (q, 2H, H-2′, *J* = 7.2 Hz), 2.32–2.51 (m, 16H, H-3′, H-4′, H-5′, H-9″, and H-1‴), 2.66 (t, 2H, H-1′, *J* = 7.3 Hz), 3.10 (s, 2H, H-6′), 3.50–3.60 (m, 6H, H-8″, and H-2‴), 6.89 (td, 1H, H-6, *J_o_* = 9.2 Hz, *J_m_* = 2.5 Hz), 7.20 (d, 1H, H-2, *J* = 2.0 Hz), 7.25 (dd, 1H, H-7, *J_o_* = 10.1 Hz, *J_m_* = 2.5 Hz), 7.29–7.40 (m, 2H, H-4 and H-4″), 7.49 (dd, 1H, H-3″, *J_o_* = 9.5 Hz, *J_m_* = 3.0 Hz), 8.53 (dd, 1H, H-6″, *J_o_* = 9.2 Hz, *J_m_* = 5.4 Hz), 8.69 (t, 1H, H-7″, *J* = 5.4 Hz), 10.89 (s, 1H, H-1), and 11.62 (br. s, 1H, H-7′) ppm. ^13^C-NMR (DMSO-*d*_6_): δ 22.2, 26.9, 36.4, 52.4 (2X), 52.9 (2X), 53.2 (2X), 57, 57.4, 61.7, 66.1 (2X), 102.9 (d, ^2^*J*_C-F_ = 22.3 Hz), 108.8 (d, ^2′^*J*_C-F_ = 26.3 Hz), 112.1 (d, ^3^*J*_C-F_ = 9.9 Hz), 114.5 (d, ^2″^*J*_C-F_ = 23.8 Hz), 114.6 (d, ^4^*J*_C-F_ = 5 Hz), 118 (d, ^2‴^*J*_C-F_ = 22 Hz), 122.1 (d, ^3′^*J*_C-F_ = 7 Hz), 123.8 (d, ^3″^*J*_C-F_ = 6.2 Hz), 124.3, 127.4 (d, ^3‴^*J*_C–F_ = 9.5 Hz), 132.9, 134.4 (d, ^4′^*J*_C-F_ = 2.2 Hz), 152.4 (d, ^1^*J*_C–F_ = 231 Hz), 156.5 (d, ^1′^*J*_C-F_ = 223 Hz), 166.3, and 169 ppm. HRMS: (EI) Calculated for C_30_H_38_F_2_N_6_O_3_ (M^+^) = 569.3051. Found: 569.3048.

##### 4-Fluoro-2-(2-{4-[3-(5-bromo-1*H*-3-indolyl)-propyl]-1-piperazinyl}-acetylamino)-*N*-(2-morpholin-4-yl-ethyl) Benzamide (**13e**)

5-Bromo-3-(3-piperazin-1-yl-propyl)-1*H*-indole **9c** (187 mg; 0.58 mmol), 2-(2-chloro-acetylamino)-4- fluoro-*N*-(2-morpholin-4-yl-ethyl)-benzamide **12a** (199 mg; 0.58 mmol), and anhydrous K_2_CO_3_ (80 mg; 0.58 mmol), to afford **13e** (236 mg; 66% yield) as a yellow light solid. m.p.: 92.1 -93.8 °C; ^1^H-NMR (DMSO-*d*_6_): δ 1.78 (q, 2H, H-2′, *J* = 7.4 Hz), 2.35 (t, 2H, H-3′, *J* = 6.9 Hz), 2.38–2.52 (m, 14H, H-4′, H-5′, H-9″ and H-1‴), 2.67 (t, 2H, H-1′, *J* = 7.3 Hz), 3.13 (s, 2H, H-6′), 3.40–3.42 (m, 2H, H-8″), 3.54 (t, 4H, H-2‴, *J* = 4.5 Hz), 7.0 (td, 1H, H-5″, *J_o_* = 8.3 Hz, *J_m_* = 2.8 Hz), 7.12–7.20 (m, 2H, H-2, and H-6), 7.31 (d, 1H, H-7, *J* = 8.6 Hz), 7.62 (d, 1H, H-4, *J* = 1.7 Hz), 7.77 (dd, 1H, H-3″, *J_o_* = 8.8 Hz, *J_m_* = 6.6 Hz), 8.39 (dd, 1H, H-6″, *J_o_* = 12.3 Hz, *J_m_* = 2.7 Hz), 8.70 (t, 1H, H-7″, *J* = 5.4 Hz), 11.1 (s, 1H, H-1), and 12.1 (br. s, 1H, H-7′) ppm. ^13^C-NMR (DMSO-*d*_6_): δ 22.8, 27.4, 36.9, 52.6 (2X), 52.7 (2X), 53.7 (2X), 56.2, 57.6, 62.2, 66.6 (2X), 107.1 (d, ^2^*J*_C-F_ = 26.5 Hz), 109.7 (d, ^2′^*J*_C-F_ = 22.7 Hz), 111.2, 113.3, 118.6 (d, ^4^*J*_C-F_ = 4.9 Hz), 118.7, 121.6, 123.7, 124.4, 129.6, 130.8 (d, ^3^*J*_C-F_ = 9.9 Hz), 135.4, 140.7 (d, ^3′^*J*_C-F_ = 12.2 Hz), 163.8 (d, ^1^*J*_C-F_ = 246 Hz), 167.3, and 170.4 ppm. HRMS: (EI) Calculated for C_30_H_38_BrFN_6_O_3_ (M^+^) = 629.2251. Found: 629.2246.

##### 4-Fluoro-3-(2-{4-[3-(5-bromo-1*H*-3-indolyl)-propyl]-1-piperazinyl}-acetylamino)-*N*-(2-morpholin-4-yl-ethyl) Benzamide (**13f**)

5-Bromo-3-(3-piperazin-1-yl-propyl)-1*H*-indole **9c** (187 mg; 0.58 mmol), 3-(2-chloro-acetylamino)- 4-fluoro-*N*-(2-morpholin-4-yl-ethyl)-benzamide **12b** (199 mg; 0.58 mmol), and anhydrous K_2_CO_3_ (80 mg; 0.58 mmol), to afford **13f** (193 mg; 54% yield) as a yellow light solid. m.p.: 85.2–86.8 °C; ^1^H-NMR (DMSO-*d*_6_): δ 1.78 (q, 2H, H-2′, *J* = 6.5 Hz), 2.30–2.55 (m, 16H, H-3′, H-4′, H-5′, H-9″, and H-1‴), 2.68 (t, 2H, H-1′, *J* = 7.3 Hz), 3.20 (s, 2H, H-6′), 3.40–3.44 (m, 2H, H-8″), 3.57 (t, 4H, H-2‴, *J* = 4.4 Hz), 7.13–7.21 (m, 2H, H-2, and H-6), 7.31 (d, 1H, H-7, *J* = 10.1 Hz), 7.34–7.42 (m, 1H, H-5″), 7.59–7.67 (m, 1H, H-2″), 7.62 (d, 1H, H-4, *J* = 1.6 Hz), 8.37–8.52 (m, 2H, H-6″ and H-7″), 9.69 (br. s, 1H, H-7′), and 11.0 (s, 1H, H-1) ppm. ^13^C-NMR (DMSO-*d*_6_): δ 22.2, 26.9, 36.4, 52.4 (2X), 52.9 (2X), 53.2 (2X), 57, 57.4, 61.7, 66.1 (2X), 111.3, 113.8, 114.7, 115.6 (d, ^2^*J*_C-F_ = 21 Hz), 121.1, 123.1, 123.28 (d, ^4^*J*_C-F_ = 2.9 Hz), 123.7, 124.5 (d, ^3^*J*_C-F_ = 9.5 Hz), 126.2 (d, ^2′^*J*_C-F_ = 11 Hz), 129.7, 131.63 (d, ^3′^*J*_C-F_ = 3.7 Hz), 135.7, 155.3 (d, ^1^*J*_C-F_ = 248 Hz), 165.6, and 169 ppm. HRMS: (EI) Calculated for C_30_H_38_BrFN_6_O_3_ (M^+^) = 629.2251. Found: 629.2250.

##### 2-Fluoro-5-(2-{4-[3-(5-bromo-1*H*-3-indolyl)-propyl]-1-piperazinyl}-acetylamino)-*N*-(2-morpholin-4-yl-ethyl) Benzamide (**13g**)

5-Bromo-3-(3-piperazin-1-yl-propyl)-1*H*-indole **9c** (187 mg; 0.58 mmol), 5-(2-chloro-acetylamino)-2- fluoro-*N*-(2-morpholin-4-yl-ethyl)-benzamide **12d** (199 mg; 0.58 mmol), and anhydrous K_2_CO_3_ (80 mg; 0.58 mmol), to afford **13g** (249 mg; 70% yield) as a yellow light solid. m.p.: 73.2–74.5 °C; ^1^H-NMR (DMSO-*d*_6_): δ 1.79 (q, 2H, H-2′, *J* = 7.2 Hz), 2.32–2.51 (m, 16 H, H-3′, H-4′, H-5′, H-9″, and H-1‴), 2.66 (t, 2H, H-1′, *J* = 7.3 Hz), 3.10 (s, 2H, H-6′), 3.50–3.60 (m, 6H, H-8″, and H-2‴), 6.89 (td, 1H, H-6, *J_o_* = 9.2 Hz, *J_m_* = 2.5 Hz), 7.20 (d, 1H, H-2, *J* = 2.0 Hz), 7.25 (dd, 1H, H-7, *J_o_* = 10.1 Hz, *J_m_* = 2.5 Hz), 7.29–7.40 (m, 2H, H-4, and H6″), 7.49 (dd, 1H, H-4″, *J_o_* = 9.5 Hz, *J_m_* = 3.0 Hz), 8.53 (dd, 1H, H-3″, *J_o_* = 9.2 Hz, *J_m_* = 5.4 Hz), 8.69 (t, 1H, H-7″, *J* = 5.4 Hz), 10.89 (s, 1H, H-1), and 11.62 (br. s, 1H, H-7′) ppm. ^13^C-NMR (DMSO-*d*_6_): δ 22.4, 27.5, 36.9, 52.9 (2X), 53 (2X), 53.6 (2X), 574, 57.6, 62.1, 66.7 (2X), 111.3, 113.8, 114.7, 116.7 (d, ^2^*J*_C-F_ = 23.5 Hz), 121.1, 121.4 (d, ^3^*J*_C-F_ = 3.7 Hz), 123.6 (d, ^3′^*J*_C-F_ = 7.3 Hz), 123.7, 124.2 (d, ^2′^*J*_C-F_ = 15.4 Hz), 124.5, 129.6, 135.3, 135.4 (d, ^4^*J*_C-F_ = 2.2 Hz), 155.4 (d, ^1^*J*_C-F_ = 245 Hz), 163.7, and 168.9 ppm. HRMS: (EI) Calculated for C_30_H_38_BrFN_6_O_3_ (M^+^) = 629.2251. Found: 629.2248.

##### 5-Fluoro-2-(2-{4-[3-(5-bromo-1*H*-3-indolyl)-propyl]-1-piperazinyl}-acetylamino)-*N*-(2-morpholin-4-yl-ethyl) Benzamide (**13h**)

5-Bromo-3-(3-piperazin-1-yl-propyl)-1*H*-indole **9c** (187 mg; 0.58 mmol), 2-(2-chloro-acetylamino)-5- fluoro-*N*-(2-morpholin-4-yl-ethyl)-benzamide **12c** (199 mg; 0.58 mmol), and anhydrous K_2_CO_3_ (80 mg; 0.58 mmol), to afford **13h** (303 mg; 85% yield) as a yellow light solid. m.p.: 73.8–74.7 °C; ^1^H-NMR (DMSO-*d*_6_): δ 1.71–1.84 (m, 2H, H-2′), 2.25–2.56 (m, 16H, H-3′, H-4′, H-5′, H-9″, and H-1‴), 2.67 (t, 2H, H-1′, *J* = 7.2 Hz), 3.14 (s, 2H, H-6′), 3.40–3.43 (m, 2H, H-8″), 3.57 (t, 4H, H-2‴, *J* = 4.5 Hz), 7.13–7.27 (m, 3H, H-2, and H-6 and H-3″), 7.31 (d, 1H, H-7, *J* = 8.5 Hz), 7.69 (d, 1H, H-4, *J* = 1.7 Hz), 7.73–7.81 (m, 1H, H-6″), 7.92 (dd, 1H, H-4″, *J_o_* = 6.4 Hz, *J_m_* = 2.7 Hz), 8.15–8.22 (m, 1H, H-7″), 9.9 (br. s, 1H, H-7′), and 11 (s, 1H, H-1) ppm. ^13^C-NMR (DMSO-*d*_6_): δ 22.4, 27.5, 36.9, 52.8 (2X), 53.3 (2X), 53.7 (2X), 57.5, 57.7, 62.2, 66.6 (2X), 111.3, 113.8, 114.7, 115.1 (d, ^2^*J*_C-F_ = 24.2 Hz), 118.5 (d, ^2′^*J*_C-F_ = 21.3 Hz), 121.1, 122.6 (d, ^3^*J*_C-F_ = 7.3 Hz), 123.7, 124.3 (d, ^3′^*J*_C-F_ = 5.9 Hz), 124.4, 129.6, 134.9 (d, ^4^*J*_C-F_ = 2.2 Hz), 135.4, 157.3 (d, ^1^*J*_C-F_ = 241 Hz), 166.9, and 169.5 ppm. HRMS: (EI) Calculated for C_30_H_38_BrFN_6_O_3_ (M^+^) = 629.2251. Found: 629.2249.

##### 4-Fluoro-2-(2-{4-[3-(1*H*-3-indolyl)-propyl]-1-piperazinyl}-acetylamino)-*N*-(2-morpholin-4-yl-ethyl) Benzamide (**13i**)

3-(3-Piperazin-1-yl-propyl)-1*H*-indole **9a** (150 mg; 0.62 mmol), 2-(2-chloro-acetylamino)-4-fluoro- *N*-(2-morpholin-4-yl-ethyl)-benzamide **12a** (213 mg; 0.62 mmol), and anhydrous K_2_CO_3_ (86 mg; 0.62 mmol), to afford **13i** (249 mg; 75% yield) as a yellow light solid. m.p.: 80.5–81.8 °C; ^1^H-NMR (DMSO-*d*_6_): δ 1.86 (m, 2H, H-2′), 2.25–2.49 (m, 12H, H-3′, H-4′, H-9″and H-1‴), 2.58 (m, 4H, H-5′), 2.7 (t, 2H, H-1′, *J* = 7.4 Hz), 3.15 (s, 2H, H-6′), 3.35–3.40 (m, 2H, H-8″), 3.54 (t, 4H, H-2‴, *J* = 4.3 Hz), 6.93–7.09 (m, 3H, H-5 and H-6 and H-5″), 7.12 (d, 1H, H-2, *J* = 1 Hz), 7.33 (d, 1H, H-7, *J* = 8 Hz), 7.51 (d, 1H, H-4, *J* = 7.8 Hz), 7.76 (dd, 1H, H-3″, *J_o_* = 8.6 Hz, *J_m_* = 6.7 Hz), 8.39 (dd, 1H, H-6″, *J_o_* = 12.3 Hz, *J_m_* = 2.6 Hz), 8.68 (t, 1H, H-7″, *J* = 5 Hz), 10.8 (s, 1H, H-1), and 12.1 (br. s, 1H, H-7′) ppm. ^13^C-NMR (DMSO-*d*_6_): δ 22.5, 27.7, 36.9, 52.9 (2X), 53.5 (2X), 53.7 (2X), 55.4, 57.6, 62.3, 66.6 (2X), 107 (d, ^2^*J*_C-F_ = 28 Hz), 109.7 (d, ^2′^*J*_C-F_ = 22 Hz), 111.8, 114.5, 118.6 (d, ^4^*J*_C-F_ = 2.9 Hz), 121.3, 122.6, 127.6, 130.8 (d, ^3^*J*_C-F_ = 10.9 Hz), 134, 136.8, 140.8 (d, ^3′^*J*_C-F_ = 12.5 Hz), 141.4, 163.8 (d, ^1^*J*_C-F_ = 245 Hz), 167.3, and 170.3 ppm. HRMS: (EI) Calculated for C_30_H_39_FN_6_O_3_ (M^+^) = 551.3145. Found: 551.3139.

##### 4-Fluoro-3-(2-{4-[3-(1*H*-3-indolyl)-propyl]-1-piperazinyl}-acetylamino)-*N*-(2-morpholin-4-yl-ethyl) Benzamide (**13j**)

3-(3-Piperazin-1-yl-propyl)-1*H*-indole **9a** (150 mg; 0.62 mmol), 3-(2-chloro-acetylamino)-4- fluoro-*N*-(2-morpholin-4-yl-ethyl)-benzamide **12b** (213 mg; 0.62 mmol), and anhydrous K_2_CO_3_ (86 mg; 0.62 mmol), to afford **13j** (268 mg; 81% yield) as a yellow light solid. m.p.: 70.2–71.9 °C; ^1^H-NMR (DMSO-*d*_6_): δ 1.81 (q, 2H, H-2′, *J* = 7.5 Hz), 2.21–2.53 (m, 16H, H-3′, H-4′, H-5′, H-9″, and H-1‴), 2.7 (t, 2H, H-1′, *J* = 7.4 Hz), 3.19 (s, 2H, H-6′), 3.38–3.40 (m, 2H, H-8″), 3.57 (t, 4H, H-2‴, *J* = 4.5 Hz), 6.96 (td, 1H, H-5 or H-6, *J_o_* = 7.5 Hz, *J_m_* = 1 Hz), 7.06 (td, 1H, H-6 or H-5, *J_o_* = 7.4 Hz, *J_m_* = 1 Hz), 7.11 (d, 1H, H-2, *J* = 2.1 Hz), 7.32–7.40 (m, 2H, H-7 and H-5″), 7.51 (d, 1H, H-4, *J* = 7.7 Hz), 7.61–7.66 (m, 1H, H-2″), 8.40–8.52 (m, 2H, H-6″ and H-7″), 9.69 (br. s, 1H, H-7′), and 10.8 (s, 1H, H-1) ppm. ^13^C-NMR (DMSO-*d*_6_): δ 22.4, 27.1, 36.6, 52.8 (2X), 52.9 (2X), 53.2 (2X), 57.2, 57.5, 61.1, 66.2 (2X), 111.3, 114.3, 115.1 (d, ^2^*J*_C-F_ = 20.1 Hz), 118.1 (d, ^3^*J*_C-F_ = 10.6 Hz), 120.7, 122.1, 122.4, 123.0, 124.0 (d, ^3′^*J*_C-F_ = 8.1 Hz), 125.7 (d, ^2′^*J*_C-F_ = 11.8 Hz), 127.2, 131.1 (d, ^4^*J*_C-F_ = 3.3 Hz), 136.3, 154.7 (d, ^1^*J*_C-F_ = 249 Hz), 165.1, and 168.5 ppm. HRMS: (EI) Calculated for C_30_H_39_FN_6_O_3_ (M^+^) = 551.3145. Found: 551.3140.

##### 2-Fluoro-5-(2-{4-[3-(1*H*-3-indolyl)-propyl]-1-piperazinyl}-acetylamino)-*N*-(2-morpholin-4-yl-ethyl) Benzamide (**13k**)

3-(3-Piperazin-1-yl-propyl)-1*H*-indole **9a** (150 mg; 0.62 mmol), 5-(2-chloro-acetylamino)-2- fluoro-*N*-(2-morpholin-4-yl-ethyl)-benzamide **12d** (213 mg; 0.62 mmol), and anhydrous K_2_CO_3_ (86 mg; 0.62 mmol), to afford **13k** (200 mg; 60.7% yield) as a yellow light solid. m.p.: 69.7–71.5 °C; ^1^H-NMR (DMSO-*d*_6_): δ 1.81 (q, 2H, H-2′, *J* = 6.8 Hz), 2.25–2.54 (m, 16H, H-3′, H-4′, H-5′, H-9″, and H-1‴), 2.7 (t, 2H, H-1′, *J* = 7.2 Hz), 3.13 (s, 2H, H-6′), 3.39 (q, 2H, H-8″, *J* = 6.4 Hz), 3.58 (t, 4H, H-2‴, *J* = 4.2 Hz), 6.96 (t, 1H, H-5 or H-6, *J* = 7.2 Hz), 7.06 (t, 1H, H-6 or H-5, *J* = 6.9 Hz), 7.11 (d, 1H, H-2, *J* = 1.2 Hz), 7.24 (t, 1H, H-3″, *J* = 9.8 Hz), 7.34 (d, 1H, H-4, *J* = 8.0 Hz), 7.51 (d, 1H, H-7, *J* = 7.7 Hz), 7.76–7.81 (m, 1H, H-6″), 7.94 (dd, 1H, H-4″, *J_o_* = 6.3 Hz, *J_m_* = 2.6 Hz), 8.15–8.22 (m, 1H, H-7″), 9.90 (br. s, 1H, H-7′), and 10.8 (s, 1H, H-1) ppm. ^13^C-NMR (DMSO-*d*_6_): δ 22.9, 27.5, 37.0, 53.0 (2X), 53.2 (2X), 53.7 (2X), 57.4, 57.9, 62.2, 66.7 (2X), 111.8, 114.8, 116.7 (d, ^2^*J*_C-F_ = 24.2 Hz), 118.5, 118.7, 121.2, 121.4 (d, ^4^*J*_C-F_ = 2.2 Hz), 112.6, 123.6 (d, ^3^*J*_C-F_ = 8.1 Hz), 124.1 (d, ^2′^*J*_C-F_ = 16.1 Hz), 127.7, 135.4 (d, ^3^*J*_C-F_ = 2.9 Hz), 136.8, 155.4 (d, ^1^*J*_C-F_ = 245 Hz), 163.7, and 168.9 ppm. HRMS: (EI) Calculated for C_30_H_39_FN_6_O_3_ (M^+^) = 551.3145. Found: 551.3142.

##### 5-Fluoro-2-(2-{4-[3-(1*H*-3-indolyl)-propyl]-1-piperazinyl}-acetylamino)-*N*-(2-morpholin-4-yl-ethyl) Benzamide (**13l**)

3-(3-Piperazin-1-yl-propyl)-1*H*-indole **9a** (200 mg; 0.82 mmol), 2-(2-chloro-acetylamino)-5- fluoro-*N*-(2-morpholin-4-yl-ethyl)-benzamide **12c** (281 mg; 0.82 mmol), and anhydrous K_2_CO_3_ (113 mg; 0.82 mmol), to afford **13l** (263 mg; 60% yield) as a yellow light solid. m.p.: 68.1–69.6 °C; ^1^H-NMR (DMSO-*d*_6_): δ 1.80 (q, 2H, H-2′, *J* = 7.6 Hz), 2.30–2.52 (m, 16H, H-3′, H-4′, H-5′, H-9″ and H-1‴), 2.69 (t, 2H, H-1′, *J* = 7.4 Hz), 3.18 (s, 2H, H-6′), 3.70–3.41 (m, 2H, H-8″), 3.56 (t, 4H, H-2‴, *J* = 4.5 Hz), 6.96 (td, 1H, H-5 or H-6, *J_o_* = 7.8 Hz, *J_m_* = 1.0 Hz), 7.05 (td, 1H, H-6 or H-5, *J_o_* = 7 Hz, J*_m_*= 1.1 Hz), 7.11 (d, 1H, H-2, *J* = 2.1 Hz), 7.33 (t, 1H, H-4″, *J* = 8.4 Hz), 7.39 (d, 1H, H-4, *J* = 8.7 Hz), 7.50 (d, 1H, H-7, *J* = 7.8 Hz), 7.59–7.66 (m, 1H, H-3″), 8.40–8.48 (m, 2H, H-6″ and H-7″), 9.68 (br. s, 1H, H-7′), and 10.75 (s, 1H, H-1) ppm. ^13^C-NMR (DMSO-*d*_6_): δ 22.9, 27.7, 37.1, 53.3 (2X), 53.4 (2X), 53.8 (2X), 57.8, 58.1, 61.6, 66.7 (2X), 111.7, 113.4, 114.9, 115.6 (d, ^2^*J*_C-F_ = 19.4 Hz), 118.6 (d, ^2′^*J*_C-F_ = 17.7 Hz), 121.2, 122.6, 123.0, 124.5 (d, ^3^*J*_C-F_ = 8.3 Hz),126.2 (d, ^3′^*J*_C-F_ = 11.6 Hz), 127.7, 131.63 (d, ^4^*J*_C-F_ = 2.8 Hz), 136.7, 155.3 (d, ^1^*J*_C-F_ = 249 Hz), 165.5, and 169.1 ppm. HRMS: (EI) Calculated for C_30_H_39_FN_6_O_3_ (M^+^) = 551.3145. Found: 551.3141.

### 3.2. Biological Assay

#### 3.2.1. Reagents

[^3^H]Paroxetine (20.8 Ci/mmol; Code NET869), [^3^H]-Methylspiperone (specific activity 64.1 Ci/mmol; NET856), membrane from clonal cell line HEK-293 that overexpresses SERT (Code: RBHSTM400UA), and membrane from CHO-K1 clonal cell line that overexpresses D_2_ receptor (Code: RBHD2CM400UA) were purchased from Perkin-Elmer (Boston, MA, USA). Fluoxetine and haloperidol were purchased from Sigma-Aldrich (St. Louis, MO, USA). All other reagents used were of analytical grade.

#### 3.2.2. SERT Binding

To determine the binding of all compounds at SERT, competitive binding assays were performed according to previously reported procedures with some modifications [36]. Briefly, assays were carried out in a total volume of 0.5 mL containing 9 µg protein of membrane from a clonal cell line HEK-293 that overexpresses SERT, 50 mM Tris buffer, pH 7.4, 120 mM NaCl, 5 mM KCl, 2 nM [^3^H]-paroxetine (specific activity 20.8 Ci/mmol, PerkinElmer), and the compounds to be tested at different concentrations (10^−9^–10^−4^ M). After 1 h at 27 °C, incubations were stopped by rapid filtration through Whatman GF/C filters presoaked in 0.5% polyethyleneimine, which were washed five times with 3 mL of ice-cold buffer, dried, and put in Eppendorf tubes with scintillation liquid. Radioactivity was counted by a liquid scintillation counter (MicroBeta 2450 microplate counter, PerkinElmer). Control curve was performed with fluoxetine in the same experimental conditions. Non-specific binding was determined with 10 µM fluoxetine.

#### 3.2.3. D_2_ Receptor Binding

To determine the binding of all compounds at D_2_ receptor, competitive binding assays were performed according to provider indications with some modifications. Briefly, assays were carried out in a total volume of 0.5 mL containing 3 µg protein of membrane from a CHO-K1 clonal cell line that overexpresses D_2_ receptor, 50 mM Tris buffer, pH 7.4, 120 mM NaCl, 5 mM KCl, 5 mM MgCl_2_, 1 mM EDTA, 0.5 nM [^3^H]-methylspiperone (specific activity 64.1 Ci/mmol, PerkinElmer), and the compounds to be tested at different concentrations (10^−9^–10^−4^ M). After 2 h at 27 °C, incubations were stopped by rapid filtration through Whatman GF/C filters presoaked in 0.5% polyethyleneimine, which were washed five times with 3 mL of ice-cold wash buffer (50 mM Tris buffer, pH 7.4, 154 mM NaCl), dried, and put in Eppendorf tubes with scintillation liquid. Radioactivity was counted as described before. Control curve was performed with haloperidol in the same conditions. Non-specific binding was determined with 10 µM haloperidol.

Analysis of data: All curves were fitted using the sigmoidal dose–response inhibition curve (variable slope) equation built into GraphPad PRISM 5.01 (GraphPad Software Inc., San Diego, CA, USA). The analysis gives the IC_50_ value (i.e., the drug concentration inhibiting specific binding by 50%) to calculate Ki (affinity constant) by the Cheng–Prussof equation (Ki = IC_50_/(1 + ([radioligand]/Kd (radioligand))). The Kd values used correspond to 0.13 nM to [^3^H] paroxetine on SERT [50], and 0.1 nM to [^3^H]-methylspiperone on D_2_ (provided by the manufacturer). The IC_50_ and Ki values correspond to the results of three independent experiments, each in triplicate. All data are expressed as the mean ± SEM.

#### 3.2.4. MAO-A Inhibition

All experimental procedures were approved by the Ethics Committee of the University of Santiago de Chile and the Science Council (FONDECYT) of Chile and followed internationally accepted guidelines (NIH Guide for the Care and Use of Laboratory Animals).

The effects of the compounds on rat MAO-A activity were studied following a previously reported methodology [50,51], using a crude rat brain mitochondrial suspension as a source of enzyme. Serotonin (100 µM) was used as the selective substrate for MAO-A. This compound and its metabolite were detected by HPLC with electrochemical detection. As an exploratory evaluation, the percentage of MAO-A inhibition in the presence of 100 µM of the different compounds was determined, with the idea of evaluating in detail those compounds showing an inhibitory activity in the range of 70–100%.

#### 3.2.5. Molecular Docking

Molecular docking studies for the two families of compounds were performed on two different protein targets (SERT and D_2_ receptor). All dockings were carried out at pH 7.4 in the crystal structures of human SERT (hSERT PDB: 5I73) [47] and human D_2_ receptor [hD_2_ PDB: 6CM4) [49]. All compounds were modelled using the Spartan’14 Software (Wavefunction, Inc. Irvine, CA) and geometry optimization calculations were carried out using the software package at the Hartree-Fock level using the 6-31G* basis set. Docking studies were performed using AutoDockv4.2 [52] software suite with Autodock Tools ADT 1.5.6 [52,53] following the standard docking procedure for rigid proteins. Grid maps were calculated using the autogrid option with a grid volume of 70 × 70 × 70 points with a grid spacing of 0.375 Å and centered on the coordinates x, y, z: 33.3 184.5; 0.565 −9.397; and 37.019 28.136 for the SERT and D_2_ receptor respectively. Docking simulations were performed with a Lamarckian genetic algorithm (LGA) and binding energies were estimated according to the internal scoring function implemented by the program; 250 independent runs per ligand were carried out with an initial population of 300 individuals. Default settings were used for all other parameters. The lowest free-energy resulting complexes were selected and further analyzed using the Visual Molecular Dynamic (VMD) visualization program [54]. Validation of the docking protocol was performed using the co-crystallized ligands (*S*)-citalopram and risperidone for SERT and D_2_, respectively.

#### 3.2.6. QSAR Methods

CoMFA and CoMSIA studies were performed with Sybyl X-1.2 software [55] installed in a Windows 10 environment on a PC with an Intel Core i7 CPU. The geometric optimization, field calculation, and charges calculation were performed as previously reported [56] (Figure S1 and Table S3 in Supplementary Material) [57]. The internal validation of the models was done by calculating the cross-validation coefficient q^2^ [58]. The models with the highest value of q^2^ were selected and then subjected to external validation [59,60,61] (Table S2). In all cases, the best models passed the validation limits [59] (Table S2). The regression graphs of each model and the tables of experimental versus calculated values are in the Supplementary Material (Table S3, Figure S2).

## 4. Conclusions

According to these results, the design of hybrid or bifunctional compounds, i.e., molecules that incorporate two pharmacophores known to act at different receptors into a single chemical entity, is an attractive approach for the development of agents having a targeted polypharmacological profile [19,62,63]. In the present work, we attempted to combine SERT effects previously demonstrated for indolylalkylpiperazine derivatives, functionalizing the parent scaffold with structural fragments of drugs with known activity upon D_2_ receptor or MAO-A. Unexpectedly, the synthesized compounds did not show, in most cases, a multitarget profile, since they exhibited a high affinity for SERT while showing almost no effect at D_2_ receptor or MAO-A. This indicates that this strategy, although plausible, requires a very fine design of the fragments to be connected and how these are going to be linked. Beyond these considerations, our results highlight the remarkable stability of the indolylpropylpiperazine skeleton as SERT ligand, which exhibits a high affinity by this target, apparently regardless of the type of the associated moiety [34,35,36]. We think that this represents an important feature for the design of polypharmacological molecules, in which an effect upon SERT is pursued. Docking and QSAR results allowed us to rationalize the high SERT affinity observed for compounds in both studied families. Thus, the presence of a halogen at the C-5 position of the indole ring and fluorine atoms at the benzoxazine (Series I) or acetanilide (Series II) moieties probably induces electronic deprotection of the corresponding aromatic rings, favoring stronger π–π interactions of these frameworks with donor aromatic residues at the binding site.

Interestingly, one of the compounds (**7n**) showed a promissory multitarget profile, being the only derivative showing a relatively high and comparable affinity for SERT and D_2_ receptor (*K*i = 84.4 and 307 nM, respectively). Even though at this time it is difficult to determine the molecular aspects underlying this pharmacological promiscuity, it is clear that for polypharmacological drugs, a similar affinity for different receptors is the most relevant characteristic, and therefore **7n** stands as a very attractive lead for further optimization.

## Figures and Tables

**Table 1:**
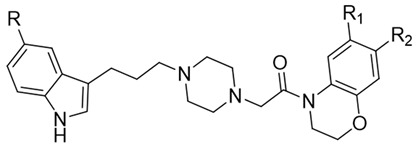
Affinities, measured as *K*i values at the serotonin transporter (SERT), D_2_ receptor, and percent of monoamine oxidase-A (MAO-A) inhibition (at 100 µM) of indolepiperazinyl benzoxazine derivatives (Series I).

Compound	R	R_1_	R_2_	SERT (nM)	D_2_ (nM)	% Inhibition MAO-A at 100 µM
Fluoxetine	-	-	-	3.15 ± 0.32	N.D.	N.D.
Haloperidol	-	-	-	N.D.	3.88 ± 0.44	N.D.
**7a**	-H	-H	-H	307.10 ± 30.46	4840 ± 68	27%
**7b**	-H	-H	-F	180.80 ± 19.97	4740 ± 131	43%
**7c**	-H	-F	-H	68.78 ± 1.02	2387 ± 163	40%
**7d**	-H	-Cl	-H	81.39 ± 4.97	1265 ± 182	18%
**7e**	-H	-Br	-H	36.57 ± 0.55	910 ± 263	38%
**7f**	-H	-OCH_3_	-H	412.20 ± 57.86	4407 ± 647	58%
**7g**	-F	-H	-H	9.63 ± 0.31	2315 ± 64	41%
**7h**	-F	-H	-F	7.61 ± 0.02	2317 ± 163	0%
**7i**	-F	-F	-H	7.51 ± 0.12	1681 ± 79	0%
**7j**	-F	-Cl	-H	12.30 ± 2.31	902 ± 4	20%
**7k**	-F	-Br	-H	5.63 ± 0.82	1041 ± 232	20%
**7l**	-F	-OCH_3_	-H	12.60 ± 1.52	3322 ± 256	52%
**7m**	-Br	-H	-H	50.18 ± 2.47	593 ± 62	30%
**7n**	-Br	-H	-F	84.44 ± 2.03	307 ± 6	23%
**7o**	-Br	-F	-H	34.89 ± 0,32	1152 ± 227	26%

Data represent the mean ± SEM of three experiments, each with triplicate samples. All *K*i values are in nM. N.D. not determined.

**Table 2:**
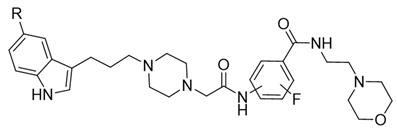
Affinities, measured as *K*i values at SERT, D_2_ receptor, and percent of MAO-A inhibition (at 100 µM) of indolepiperazinylmorpholinoethyl benzamide derivatives (Series II).

Compound	R	Position (Indolylpropyl Piperazineacetamide)	Position F	SERT (nM)	D_2_ (nM)	% Inhibition MAO-A at 100 µM
Fluoxetine	-	-	-	3.15 ± 0.32	N.D.	N.D.
Haloperidol	-	-	-	N.D.	3.88 ± 0.44	N.D.
**13a**	F	2	4	16.04 ± 0.21	5455 ± 25	19%
**13b**	F	3	4	23.78 ± 0.46	3100 ± 42	42%
**13c**	F	5	2	6.83 ± 0.19	592.60 ± 30.22	28%
**13d**	F	2	5	98.14 ± 1.24	9060 ± 29	30%
**13e**	Br	2	4	222.60 ± 16.44	5619 ± 31	39%
**13f**	Br	3	4	58.85 ± 1.14	1832 ± 33	39%
**13g**	Br	5	2	12.62 ± 0.42	765 ± 16	16%
**13h**	Br	2	5	199.60 ± 14.53	4700 ± 168	9.4%
**13i**	H	2	4	9945 ± 383	48910 ± 1279	23%
**13j**	H	3	4	109.90 ± 3.26	2339 ± 45	44%
**13k**	H	5	2	26.81 ± 0.91	4169 ± 335	34%
**13l**	H	2	5	52.16 ± 3.24	2000 ± 34	34%

Data represent the mean ± SEM of three experiments, each with triplicate samples. All *K*i values are in nM range. N.D. no determined.

## Supplementary Materials

Supplementary materials are available online. Table S1. Statistical parameters and Field combinations for CoMFA and CoMSIA. Table S2. Summary of external validation parameters for CoMFA and CoMSIA. Table S3. Experimental and predicted pKi and residual values for analyzed compounds according to CoMFA and CoMSIA. Figure S1. The superimposed structures of all compounds used in the CoMFA/CoMSIA models. Figure S2. Plots of experimental versus predicted pKi values for the training and test set molecules for CoMFA (A, B) and CoMSIA (C, D) models. Figure S3. hSERT affinity curves for compounds of Series I (**7a**, **7b**, **7c**, **7d**, **7e**, **7f**, **7g**, **7h**, **7i**, **7j**, **7k**, **7l**, **7m**, **7n**, **7o**, and fluoxetine), displaying IC_50_ values. Each determination was made in triplicate and the data were expressed as the mean ± SD. Figure S4. D2 affinity curves for compounds of Series I (**7a**, **7b**, **7c**, **7d**, **7e**, **7f**, **7g**, **7h**, **7i**, **7j**, **7k**, **7l**, **7m**, **7n**, **7o**, and haloperidol), displaying IC_50_ values. Each determination was made in triplicate and the data were expressed as the mean ± SD. Figure S5. hSERT affinity curves for compounds of Series II (**13a**, **13b**, **13c**, **13d**, **13e**, **13f**, **13g**, **13h**, **13i**, **13j**, **13k**, **13l**, and fluoxetine), displaying IC_50_ values. Each determination was made in triplicate and the data were expressed as the mean ± SD. Figure S6. D2 affinity curves for compounds of Series II (**13a**, **13b**, **13c**, **13d**, **13e**, **13f**, **13g**, **13h**, **13i**, **13j**, **13k**, **13l**, and haloperidol), displaying IC_50_ values. Each determination was made in triplicate and the data expressed as the mean ± SD.
