# Supplementary material for: Synthesis, Docking, 3-D-Qsar, and Biological Assays of Novel Indole Derivatives Targeting Serotonin Transporter, Dopamine D2 Receptor, and Mao-A Enzyme: In the Pursuit for Potential Multitarget Directed Ligands
Source: Molecules. 2020 Oct 10;25(20):4614. doi: 10.3390/molecules25204614 (PMC7594025; doi:10.3390/molecules25204614)
Supplement: Supplementary file 1 [file molecules-25-04614-s001.pdf]

Synthesis, docking, 3D-QSAR, and biological assays of novel indole derivatives targeting serotonin transporter, dopamine D2 receptor and MAO-A enzyme: in the pursuit for potential multitarget directed ligands.

Christopher Cerda-Cavieres[1], Gabriel Quiroz[2], Patricio Iturriaga-Vasquez[3], Julio Rodríguez-Lavado[1], Jazmín Alarcón-Espósito[1], Claudio Saitz[1], Carlos D. Pessoa-Mahana[4], Hery Chung[4], Ramiro Araya-Maturana[5], Jaime Mella[6,7], David Cabezas[6], Claudia Ojeda-Gómez[8], Miguel Reyes-Parada[9,10*] and Hernán Pessoa-Mahana[1*].

[1]Departamento de Química Orgánica y Fisicoquímica, Facultad de Ciencias Químicas y Farmacéuticas, Universidad de Chile, Casilla 233, Santiago, Chile.

[2]Programa de Doctorado en Farmacología, Universidad de Chile, Santiago, Chile.

[3]Departamento de Ciencias Químicas y Recursos Naturales, Facultad de Ingeniería y Ciencias, Universidad de la Frontera, Temuco, Chile.

[4]Departamento de Farmacia, Facultad de Química, Pontificia Universidad Católica de Chile, Santiago, Chile.
[5]Instituto de Química y Recursos Naturales, Universidad de Talca, Talca, Chile.

[6]Instituto de Química y Bioquímica, Facultad de Ciencias, Universidad de Valparaíso, Av. Gran Bretaña 1111, Valparaíso 2360102, Chile.

[7]Centro de Investigación Farmacopea Chilena (CIFAR), Universidad de Valparaíso, Av. Gran Bretaña 1111, Valparaíso 2360102, Chile.

[8]Colegio Instituto San Martín, Hermanos Maristas, Curicó, Chile.

[9]Centro de Investigación Biomédica y Aplicada (CIBAP), Escuela de Medicina, Facultad de Ciencias Médicas, Universidad de Santiago de Chile, Santiago, Chile.

[10]Facultad de Ciencias de la Salud, Universidad Autónoma de Chile, Talca, Chile.

Supplementary data

Table S1. Statistical parameters and Field combinations for CoMFA and CoMSIA.

Table S2. Summary of external validation parameters for CoMFA and CoMSIA.

Table S3. Experimental and predicted p*K*i and residual values for analyzed compounds according to CoMFA and CoMSIA.

Figure S1. The superimposed structures of all compounds used in the CoMFA/CoMSIA models.

Figure S2. Plots of experimental versus predicted p*K*i values for the training and test set molecules for CoMFA (**A, B**) and CoMSIA (**C, D**) models.

Figure S3. *h*SERT affinity curve of compounds of **Series I (7a**, **7b**, **7c**, **7d**, **7e**, **7f**, **7g**, **7h**, **7i**, **7j**, **7k**, **7l**, **7m**, **7n**, **7o**, and fluoxetine), displaying IC_{50} values. Each determination was made in triplicate and the data were expressed as the mean ± SD.

Figure S4. D_2 affinity curve of compounds of **Series I (7a**, **7b**, **7c**, **7d**, **7e**, **7f**, **7g**, **7h**, **7i**, **7j**, **7k**, **7l**, **7m**, **7n**, **7o**, and haloperidol), displaying IC_{50} values. Each determination was made in triplicate and the data were expressed as the mean ± SD.

Figure S5. *h*SERT affinity curve of compounds of **Series II (13a**, **13b**, **13c**, **13d**, **13e**, **13f**, **13g**, **13h**, **13i**, **13j**, **13k**, **13l**, and fluoxetine), displaying IC_{50} values. Each determination was made in triplicate and the data were expressed as the mean ± SD.

Figure S6. D_2 affinity curve of compounds of **Series II (13a**, **13b**, **13c**, **13d**, **13e**, **13f**, **13g**, **13h**, **13i**, **13j**, **13k**, **13l**, and haloperidol), displaying IC_{50} values. Each determination was made in triplicate and the data were expressed as the mean ± SD.

QSAR Statistical results

A summary of the statistical results for the best CoMFA and CoMSIA models is presented in Table S1. The best models were searched through successive field combinations. The first parameter to evaluate the statistical robustness of a QSAR model is the value of q^2, which must be greater than 0.5 q^2 is an indicator of the internal predictive capacity of a QSAR model. The external validation of the models is presented in Table S2.

Table S1. Statistical parameters and Field combinations for CoMFA and CoMSIA.[a]

								Field Contributions		
**Model**	**q^2**	**N**	**SEP**	**SEE**	**r^2_{ncv}**	**F**	**r^2**	**S**	**E**	**H**
CoMFA-SE	0.625	8	0.519	0.153	0.967	33.3	0.717	0.39	0.61	-
CoMSIA-EH	0.523	6	0.529	0.155	0.959	42.9	0.702	-	0.674	0.326

[a] q^2 = the square of the LOO cross-validation (CV) coefficient; N = the optimum number of components; SEP = standard error of prediction; SEE is the standard error of estimation of non-CV analysis; r^2_{ncv} is the square of the non CV coefficient; F is the F-test value; r^2 is the predictive r^2 for test set compounds; S, E and H are the steric, electrostatic and Hydrophobic contributions respectively.

Table S2. Summary of external validation parameters for CoMFA and CoMSIA.

**Condition**	**Parameters**	**Threshold value**	**CoMFA**	**CoMSIA**
1	q^2	>0.5	0.625	0.523
2	r^2	>0.6	0.717	0.702
3a	r_0^2	Close to value of r^2	0.999	0.999
3b	r'^2_0	Close to value of r^2	0.999	0.999
4a	k	0.85 < k < 1.15	1.016	1.006
4b	k'	0.85 < k < 1.15	0.983	0.993
5	$\|r_0^2 - r'^2_0\|$	<0.3	0.00	0.00

q^2 and r^2 are the same parameters as listed in Table 1; r_0^2 and k are the correlation coefficient between the experimental versus predicted activities for test set (*x vs y*) through the origin and the respective slope of regression; and $r_0'^2$ and k' are the correlation coefficient between the predicted versus experimental activities for test set (*y vs x*) through the origin and the respective slope of regression.

The values of experimental activity, predicted activity, and residual values for the best CoMFA and CoMSIA models are shown in Table S3.

Table S3. Experimental and predicted p*K*i and residual values for analyzed compounds according to CoMFA and CoMSIA.

		CoMFA		CoMSIA	
**Molecule**	**Exp. pKi**	**Pred. pKi**	**Residual**	**Pred. pKi**	**Residual**
**7a**	6.513	6.668	-0.16	6.663	-0.15
**7b**[t]	6.743	6.915	-0.17	6.991	-0.25
**7c**	7.163	7.003	0.16	6.952	0.21
**7d**[t]	7.089	7.201	-0.11	7.126	-0.04
**7e**	7.437	7.276	0.16	7.189	0.25
**7f**	6.385	6.549	-0.16	6.591	-0.21
**7g**	8.016	7.852	0.16	7.894	0.12
**7h**	8.119	8.104	0.02	8.223	-0.10
**7i**	8.124	8.185	-0.06	8.158	-0.03
**7j**[t]	7.910	8.384	-0.47	8.332	-0.42
**7k**	8.250	8.460	-0.21	8.397	-0.15
**7l**	7.900	7.737	0.16	7.770	0.13
**7m**[t]	7.300	7.193	0.11	7.293	0.01
**7n**[t]	7.074	7.450	-0.38	7.623	-0.55
**7o**	7.457	7.531	-0.07	7.558	-0.10
**13a**	7.795	7.814	-0.02	7.708	0.09
**13b**	7.624	7.623	0.00	7.612	0.01
**13c**	8.166	8.161	0.01	8.160	0.01
**13d**	7.008	7.029	-0.02	7.033	-0.03
**13e**	6.652	6.621	0.03	6.708	-0.06
**13f**[t]	7.230	7.288	-0.06	7.196	0.03
**13g**[t]	7.899	7.645	0.25	7.526	0.37
**13h**	6.700	6.687	0.01	6.646	0.05
**13i**	5.002	7.122	-2.12	7.172	-2.17
**13j**	6.959	6.961	0.00	6.940	0.02
**13k**[t]	7.572	7.020	0.55	6.865	0.71
**13l**	7.283	7.291	-0.01	7.349	-0.07

[t] test set compound

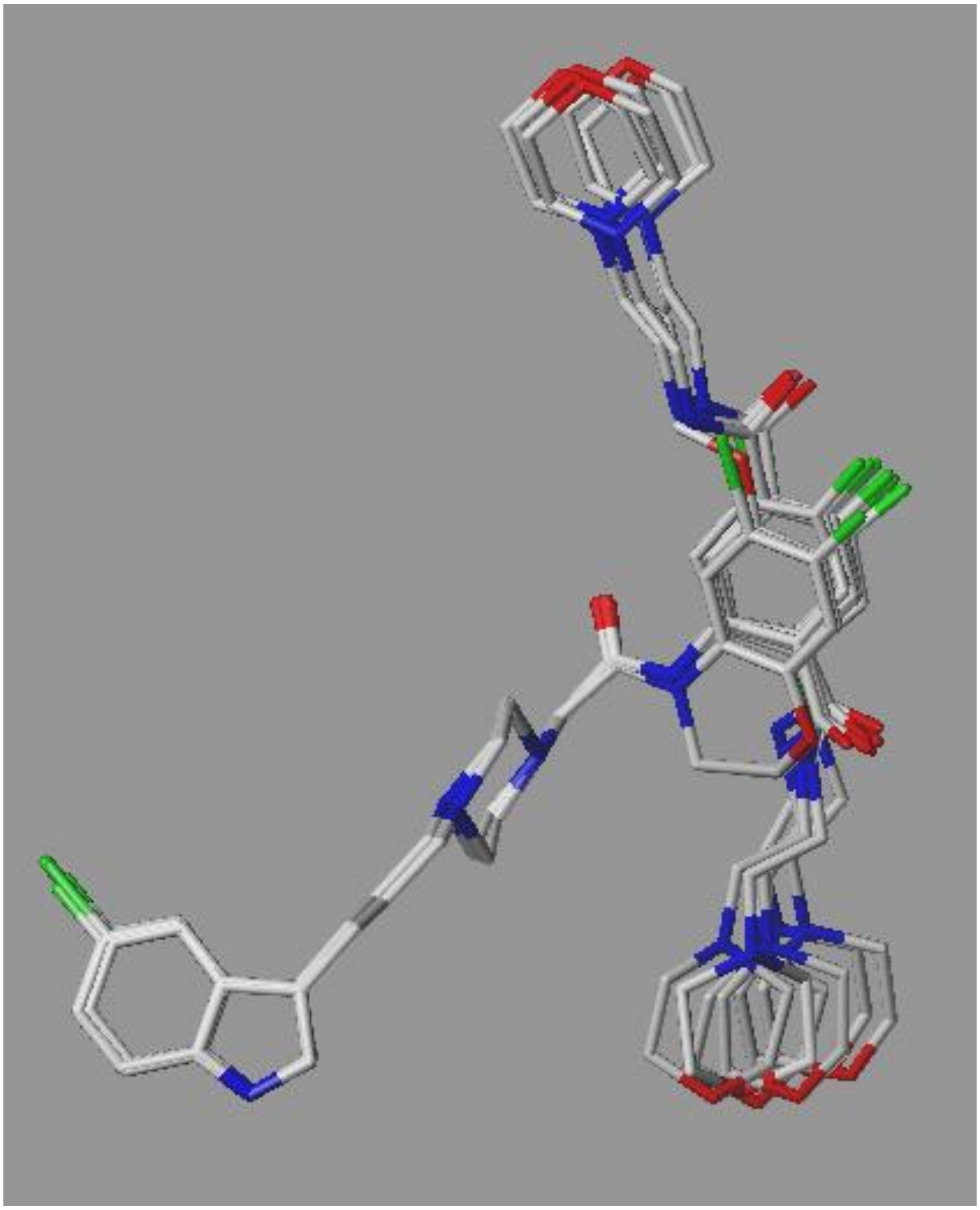

Figure S1. The superimposed structures of all compounds used in the CoMFA/CoMSIA models.

Figure S2 show the graphs of experimental versus predicted affinity for CoMFA and CoMSIA models, from which a good distribution of data along the ideal line $y = x$ is observed. Both models show a good balance in terms of predictive capacity.

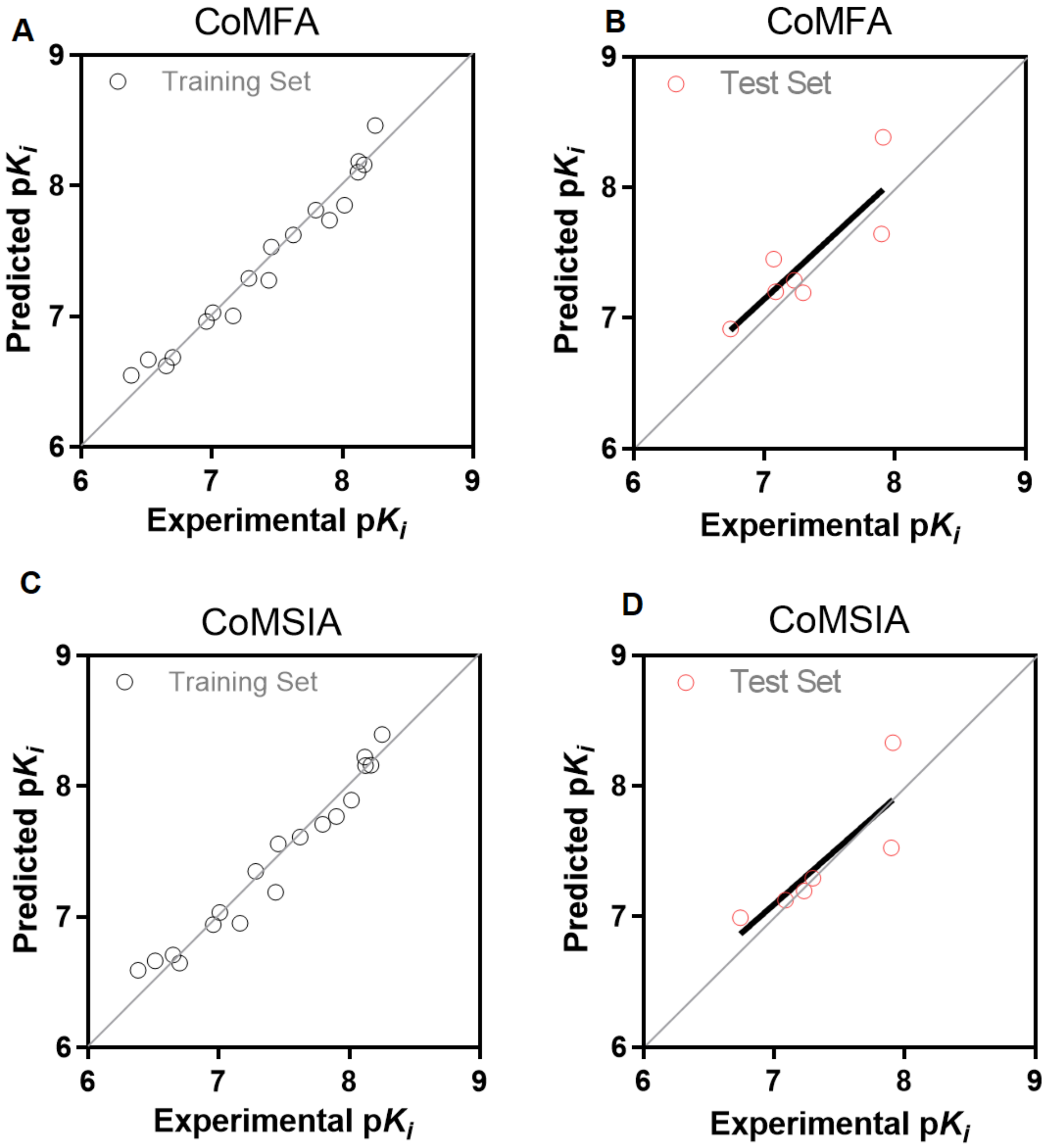

Figure S2. Plots of experimental versus predicted p*K*i values for the training and test set molecules for CoMFA (**A, B**) and CoMSIA (**C, D**) models.

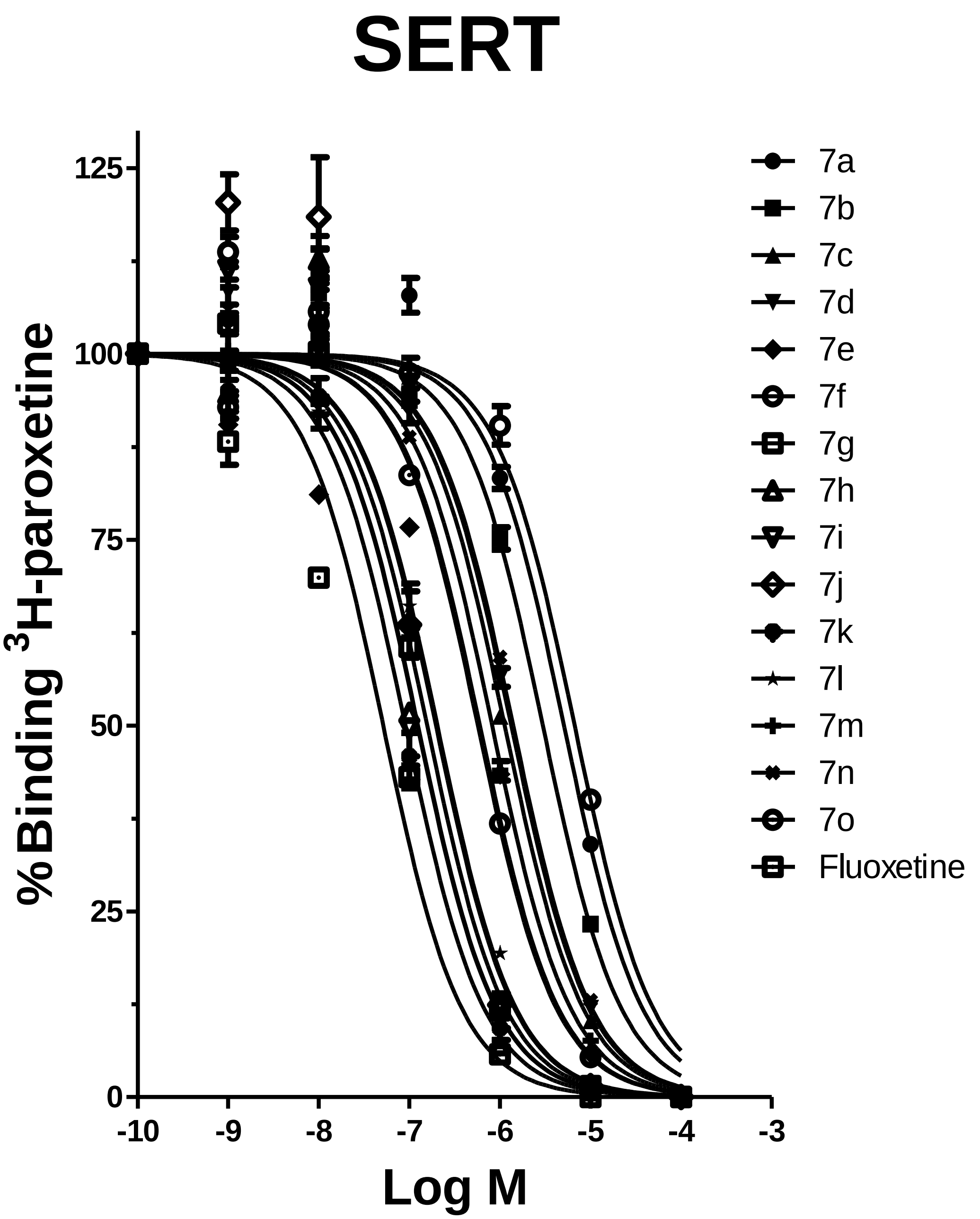

Figure S3. *h*SERT affinity curve of compounds of **Series I (7a**, **7b**, **7c**, **7d**, **7e**, **7f**, **7g**, **7h**, **7i**, **7j**, **7k**, **7l**, **7m**, **7n**, **7o**, and fluoxetine), displaying IC_{50} values. Each determination was made in triplicate and the data were expressed as the mean ± SD.

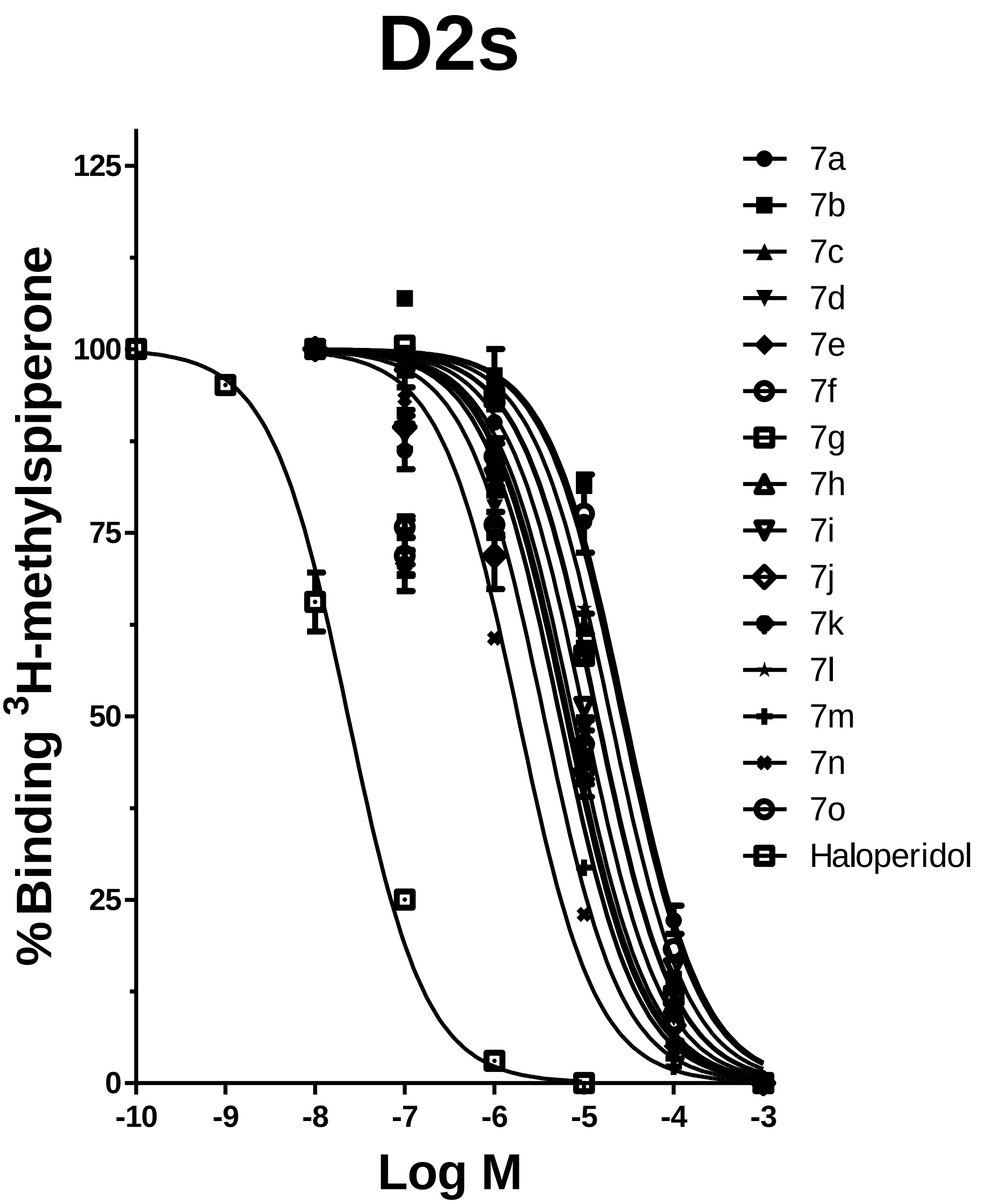

Figure S4. D_2 affinity curve of compounds of **Series I (7a**, **7b**, **7c**, **7d**, **7e**, **7f**, **7g**, **7h**, **7i**, **7j**, **7k**, **7l**, **7m**, **7n**, **7o**, and haloperidol), displaying IC_{50} values. Each determination was made in triplicate and the data were expressed as the mean ± SD.

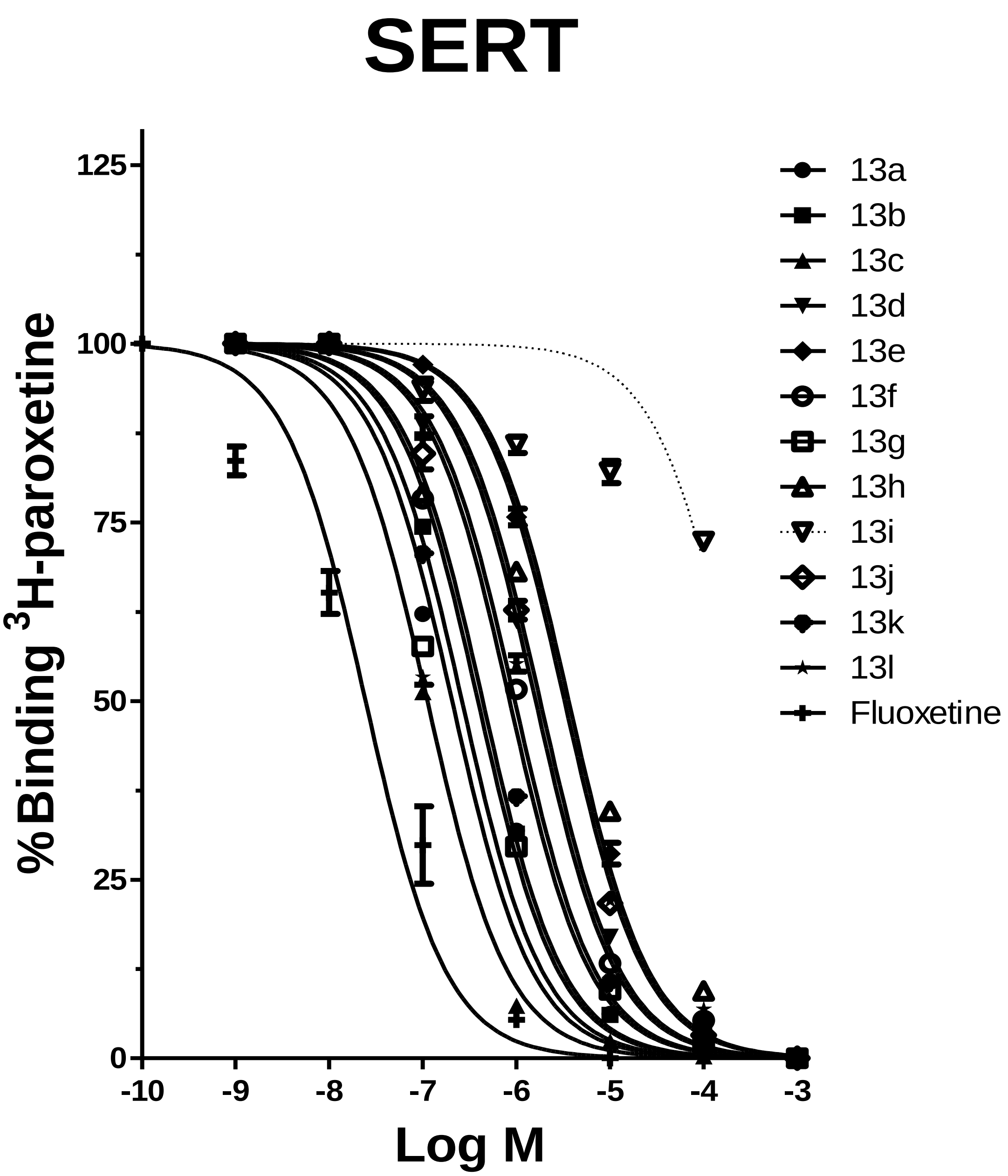

Figure S5. *h*SERT affinity curve of compounds of **Series II (13a**, **13b**, **13c**, **13d**, **13e**, **13f**, **13g**, **13h**, **13i**, **13j**, **13k**, **13l**, and fluoxetine), displaying IC_{50} values. Each determination was made in triplicate and the data were expressed as the mean ± SD.

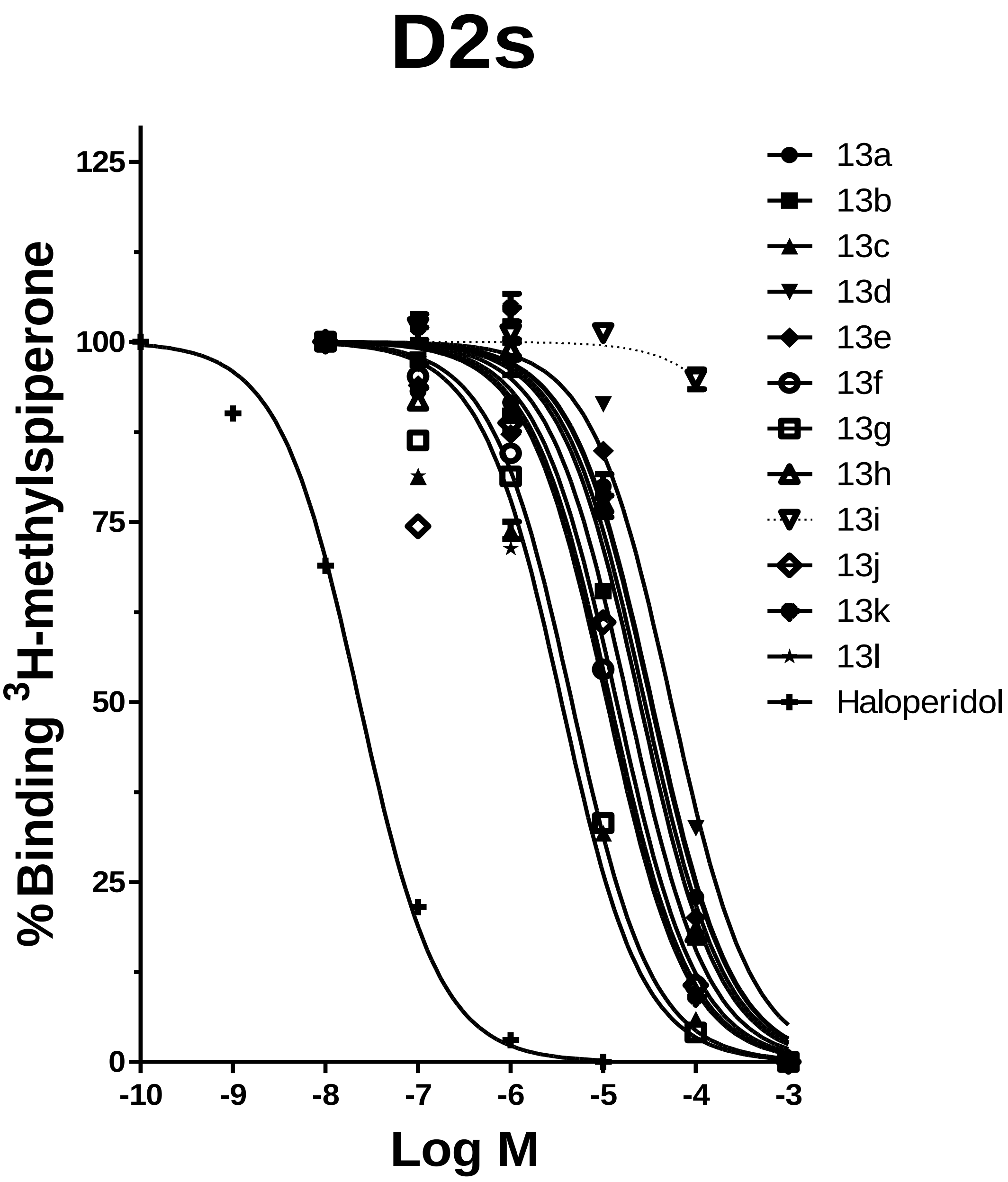

Figure S6. D_2 affinity curve of compounds of **Series II (13a**, **13b**, **13c**, **13d**, **13e**, **13f**, **13g**, **13h**, **13i**, **13j**, **13k**, **13l**, and haloperidol), displaying IC_{50} values. Each determination was made in triplicate and the data were expressed as the mean ± SD.
